# Anthocyanin and Flavonol Glycoside Metabolic Pathways Underpin Floral Color Mimicry and Contrast in a Sexually Deceptive Orchid

**DOI:** 10.3389/fpls.2022.860997

**Published:** 2022-03-23

**Authors:** Darren C. J. Wong, James Perkins, Rod Peakall

**Affiliations:** Ecology and Evolution, Research School of Biology, Australian National University, Canberra, ACT, Australia

**Keywords:** *Chiloglottis*, anthocyanin, flavonol glycoside, flower, orchids, transcriptome, sexual deception, mimicry

## Abstract

Sexually deceptive plants secure pollination by luring specific male insects as pollinators using a combination of olfactory, visual, and morphological mimicry. Flower color is a key component to this attraction, but its chemical and genetic basis remains poorly understood. *Chiloglottis trapeziformis* is a sexually deceptive orchid which has predominantly dull green-red flowers except for the central black callus projecting from the labellum lamina. The callus mimics the female of the pollinator and the stark color contrast between the black callus and dull green or red lamina is thought to enhance the visibility of the mimic. The goal of this study was to investigate the chemical composition and genetic regulation of temporal and spatial color patterns leading to visual mimicry, by integrating targeted metabolite profiling and transcriptomic analysis. Even at the very young bud stage, high levels of anthocyanins were detected in the dark callus, with peak accumulation by the mature bud stage. In contrast, anthocyanin levels in the lamina peaked as the buds opened and became reddish-green. Coordinated upregulation of multiple genes, including dihydroflavonol reductase and leucoanthocyanidin dioxygenase, and the downregulation of flavonol synthase genes (*FLS*) in the callus at the very young bud stage underpins the initial high anthocyanin levels. Conversely, within the lamina, upregulated *FLS* genes promote flavonol glycoside over anthocyanin production, with the downstream upregulation of flavonoid O-methyltransferase genes further contributing to the accumulation of methylated flavonol glycosides, whose levels peaked in the mature bud stage. Finally, the peak anthocyanin content of the reddish-green lamina of the open flower is underpinned by small increases in gene expression levels and/or differential upregulation in the lamina in select anthocyanin genes while *FLS* patterns showed little change. Differential expression of candidate genes involved in specific transport, vacuolar acidification, and photosynthetic pathways may also assist in maintaining the distinct callus and contrasting lamina color from the earliest bud stage through to the mature flower. Our findings highlight that flower color in this sexually deceptive orchid is achieved by complex tissue-specific coordinated regulation of genes and biochemical pathways across multiple developmental stages.

## Introduction

The majority of angiosperms are pollinated by animals (Ollerton et al., 2011), with floral signaling key to this crucial interaction. Flowers not only contain the reproductive structures, but also convey long-range visual and olfactory signals for pollinator attraction and the simultaneous deterrence of unwanted visitors (Raguso, 2004; Borghi et al., 2017). Brightly colored flowers are the norm in diurnally pollinated plants, ensuring visibility to pollinators against the background, with flower colors spanning the UV and visible spectrum. In addition to bright colors, many flowers display contrasting color markings, spots, and lines, which serve as “nectar guides” for pollinators, with some markings only visible to pollinators that can perceive UV light (Davies et al., 2012; Glover et al., 2013; Nadot and Carrive, 2021).

A diverse range of pigment compounds are responsible for the various colors and markings of flowers. Anthocyanins typically confer the orange, red, violet, and blue colors to flowers, while chalcones, aurones, flavonols, and flavones are often associated with different degrees of pale yellow coloration. Chlorophylls and carotenoids can further extend the color palette to include green and yellow-to-red colors (Grotewold, 2006; Tanaka and Tanaka, 2006; Tanaka et al., 2008). Importantly, the color of flowers is determined not only by the specific pigment compounds present, but also by their interactions with cellular pH, metal ions, and other co-pigments (Tanaka et al., 2008).

The anthocyanin and flavonol glycoside pathway is highly conserved across the flowering plants (Grotewold, 2006; Tanaka et al., 2008; Davies et al., 2012), and thus provides a helpful framework for any new investigation of the molecular basis of flower color. Dramatic floral color changes can be mediated by simple genetic changes affecting the regulation, expression, or function of just a few genes, or via more complex coordinated changes over many genes within the anthocyanin and flavonol pathway (Wessinger and Rausher, 2012; Tanaka and Brugliera, 2013; Zhao and Tao, 2015). Floral color changes can also be due to changes in the downstream genes that modify anthocyanins and flavonol glycosides (Morita et al., 2015). Even further downstream, changes in transport genes (Cheng et al., 2015; Zhao et al., 2020; Lu et al., 2021), and genes associated with the pH of vacuole storage (Fukada-Tanaka et al., 2000; Faraco et al., 2014) can also impact flower color. A case for non-catalytic proteins that can influence anthocyanin production, and so alter flower colors, is also known (Morita et al., 2014).

It is also now well-established that a highly conserved protein complex consisting of MYB–basic-helix-loop-helix (bHLH)–WD40 repeat (WDR) proteins (MBW) are key regulatory determinants of floral pigmentation and patterning in numerous plants (Wong et al., 2016; Allan and Espley, 2018; LaFountain and Yuan, 2021; Rodrigues et al., 2021). However, the precise components of this complex and its transcriptional control are poorly understood in non-model plant species (Davies et al., 2012; Wessinger and Rausher, 2012). Recent work has nonetheless highlighted the complexity of the anthocyanin gene regulatory network, which integrates distinct hierarchical, feedback, and repressor activities, as well as intercellular mobility of some MBW components that execute various developmental stage, tissue-specific, and stress-responsive pigmentation programs (Albert et al., 2014). Due to these complexities, it is impossible to accurately predict the pigments present and the mechanisms involved in pigmentation of a flower based on its color alone. Therefore, chemical and molecular investigations are necessary, particularly for unusually colored flowers.

Uniformly black flowers are very rare in nature, although human interest has resulted in the development of some black-flowered ornamental cultivars of dahlias, lilies, pansies, and tulips (Davies, 2008). Due to the relative rarity of black flowers, the chemical and genetic basis of black coloration in flowers remains poorly understood (Grotewold, 2006; Davies, 2008; Tanaka et al., 2008; Davies et al., 2012; Iwashina, 2015). However, it is clear that an overaccumulation of anthocyanins is largely responsible for the black colors of flowers (Markham et al., 2004; Thomas et al., 2009; Thill et al., 2012; Kellenberger et al., 2019), leaves (Hatier and Gould, 2007; Zheng et al., 2019), grains (Abdel-Aal et al., 2006; Yang et al., 2019), and fruits (Boss et al., 1996; Fan-Chiang and Wrolstad, 2006; Liu et al., 2018).

Examples of naturally black flowers include *Lisianthius nigrescens* where the uniformly dark colored petals contain extraordinary high levels of delphinidin-based anthocyanins (Markham et al., 2004). In the alpine orchid *Gymnadenia rhellicani*, higher amounts of cyanidin-based anthocyanins were observed in the petals of the black compared to red or white floral morphs (Kellenberger et al., 2019). This high anthocyanin content is linked to differential expression of an anthocyanidin synthase gene, *GrANS1* brought by loss-of-function mutation of its regulator, *GrMYB1* (Kellenberger et al., 2019). Local tissue-specific upregulation is also involved in the formation of specific dark markings and spots that generate strong contrasting colors on the flower (Martins et al., 2013, 2017; Li et al., 2014; Zhang et al., 2015). For example, in several subspecies of *Clarkia gracilis* (e.g., the basal-spotted ssp. *albicaulis* and the central-spotted ssp. *sonomensis*), the appearance of reddish-purple petal spots containing cyanidin and peonidin pigments is largely the result of spatially restricted dihydroflavonol reductase (*DFR*) expression in the early stages of flower development (Martins et al., 2013, 2017).

Competition between flavonol glycoside and anthocyanin biosynthesis is predicted to be another common mechanism underpinning variation in the spatial pattern of coloration, although the details remain poorly understood (Sheehan et al., 2016; Yuan et al., 2016). Flavonol synthase (FLS) and DFR use the same dihydroflavanol substrates, but produce flavonol and anthocyanin biosynthetic products, respectively (Tanaka and Brugliera, 2013). Flavonols are uncolored or only faintly colored compared to the vibrant colors of anthocyanins, so tissue- or region-specific coloration can be mediated by the differential expression of these two genes. For example, the absence of anthocyanins in the white areas surrounding the otherwise pink corolla of bumblebee-pollinated *Mimulus lewisii* flowers is due to high *FLS* expression, while low *FLS* expression abolishes this spatial patterning in red hummingbird-pollinated *Mimulus cardinalis* flowers (Yuan et al., 2016). Another study showed that constitutive overexpression of three distinct *FLS* from various plant species (*Rosa rugosa*, *Prunus persica*, and *Petunia hybrida*) in tobacco resulted in white flowers compared to pale pink flowers in control plants (Luo et al., 2016). Conversely, constitutive overexpression of *DFR* genes in transgenic tobacco plants led to increased anthocyanin accumulation and a deep red flower phenotype.

**FIGURE 1:**
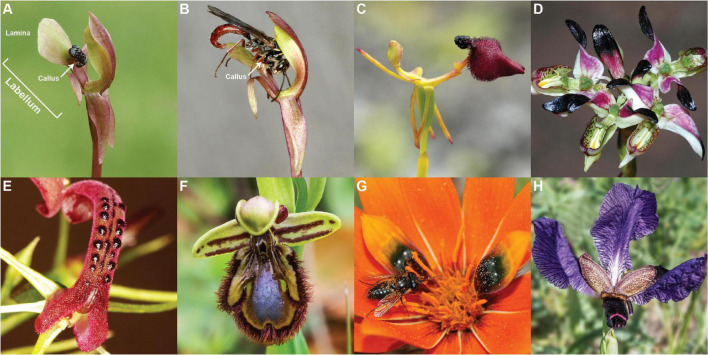
An illustration of the diversity of sexually deceptive orchid and non-orchid species. Orchids: **(A)** The floral structure of *Chiloglottis trapeziformis* with **(B)** its male pollinator *Neozeleboria cryptoides*. The arrows point to the very dark, three dimensional “callus” structure on the dull green and red labellum lamina of *Chiloglottis trapeziformis*, **(C)** *Drakaea glyptodon*, **(D)** *Disa atricapilla*, **(E)** *Cryptostylis leptochila*, and **(F)** *Ophrys speculum*. Non-orchids: **(G)** *Gorteria diffusa*, and **(H)** *Iris paradoxa*. All images have been reproduced with permission from the respective copyright holders. Please refer to the Section “Acknowledgments” for image credits.

Sexually deceptive plants secure pollination by luring specific male insects as pollinators using a combination of olfactory, visual, and morphological mimicry (Bohman et al., 2016). Within the orchids, pollination by sexual deception has evolved independently on four continents and is employed by hundreds of species spanning multiple lineages of the Orchidaceae (Peakall et al., 2020). The types of pollinators involved are also diverse, and include ants, bees and wasps (Hymenoptera), fungus gnats and other flies (Diptera), and beetles (Coleoptera) (Gaskett, 2011; Bohman et al., 2016; Peakall et al., 2020; Cohen et al., 2021; Hayashi et al., 2021). In these cases, pollination is achieved during attempted copulation with the labellum, an often highly modified petal of the orchids. Furthermore, in the multiple cases now chemically characterized, sex pheromone mimicry, often involving unusual compounds or unusual blends, is critical for the long range attraction of the pollinator (Bohman et al., 2016; Wong et al., 2017b; Peakall et al., 2020). Rather than being brightly colored, sexually deceptive orchid flowers are most often dull red and green in color, with the labellum often exhibiting starkly contrasting dark structures and markings which are thought to be visual cues that improve the insect mimicry (Figure 1, see also photos in Bohman et al., 2016; Johnson and Schiestl, 2016; Peakall et al., 2020). Outside of the Orchidaceae, there are two other known cases of pollination by sexual deception: *Gorteria diffusa* (Figure 1G) in the Asteraceae (Ellis and Johnson, 2010) and *Iris paradoxa* (Figure 1H) in the Iridaceae (Vereecken et al., 2012) both characterized by flowers that include some very dark pigmentation.

Beyond the potential role of female visual mimicry, where a close match to the color of the female of the pollinator is expected (de Jager and Peakall, 2016), the tissue-specific pigmentation patterns of sexually deceptive flowers may also provide strong color contrasts that improve detectability. For example, *Drakaea livida* shares the same male thynnine wasp pollinator as the unrelated, morphologically distinct, and differently colored *Caladenia decora*, *C. pectinata*, and *C. procera*. Yet, across this set of species, tissue-specific pigmentation provides strong achromatic contrast against a chromatic background, thus, potentially enhancing the detectability by the pollinator (Gaskett et al., 2017).

The very dark, three dimensional “calli” on the dull green and red labellum lamina of sexually deceptive *Chiloglottis* orchids are crucial to their sexual mimicry (Figure 1). In the most well-studied case of *C. trapeziformis*, the central black callus structure is the source of the unique volatile compound chiloglottone 1, which is the sex pheromone of their specific thynnine wasp pollinator, *Neozeleboria cryptoides* (Schiestl et al., 2003; Franke et al., 2009). The structure also serves as a visual and tactile mimic of the female, providing a gripping point for male wasps as they attempt to copulate with the tip of the labellum (de Jager and Peakall, 2016). Furthermore, the distance from callus to labellum tip is a close match to the length of the female wasp, and experimentally shortened or elongated labella result in significantly reduced durations of attempted copulation, indicating the importance of morphological mimicry (de Jager and Peakall, 2016, 2019). Finally, spectral reflectance measurements revealed strong similarity between the perceived color of the black callus and the female wasp abdomen in hymenopteran visual models. Both female and orchid structures are predicted to be perceived as uncolored or achromatic to male wasps, consistent with the hypothesis that the dark callus structure acts as a visual mimic of the female (de Jager and Peakall, 2016). Conversely, the surrounding lamina of the labellum is chromatic, indicating morphological adaptations to produce within-flower chromatic/achromatic contrast (Figure 1).

As the first step toward understanding the chemical and molecular basis of floral color adaptations in *Chiloglottis trapeziformis*, we leveraged targeted metabolite and transcriptome analysis of a flower tissue and developmental series to ask the following questions: (1) What anthocyanins and flavonol glycoside co-pigments are present in the flower? (2) What are the spatiotemporal patterns of anthocyanin and flavonol glycoside levels across flower development and tissue types? (3) Is the unique spatiotemporal distribution of color in the flower explained by differential expression of a few anthocyanin and flavonol glycoside pathway-related genes at specific developmental stages, or coordinated regulation at many genes and multiple stages? We propose possible mechanisms for the tissue-specific pigmentation and its regulation and discuss the evolutionary implications of these findings more broadly for *Chiloglottis* and other sexually deceptive plants.

## Materials and Methods

### Sample Collection

This study builds on a larger effort to understand the biochemistry, biosynthesis and regulation of chiloglottones, the unique semiochemicals used by sexually deceptive *Chiloglottis* orchids (Falara et al., 2013; Amarasinghe et al., 2015; Wong et al., 2017a,^2018^, ^2019^). Here, we turn attention to the chemistry and biosynthesis of flower color in *Chiloglottis trapeziformis*. Flowers at five developmental stages (very young buds, *vyb*; young bud, *yb*; mature buds, *mb*; very mature bud, *vmb*; and mature flowers open to the sun, or sun flowers, *sflw*) were sampled from the Australian National Botanic Gardens (Canberra, ACT, Australia) in September 2014. All tissues were immediately snap-frozen in liquid nitrogen at the point of collection and stored at –80°C until further use. Dissection of floral tissues into callus and labellum remains were performed in liquid N_2_.

**FIGURE 2:**
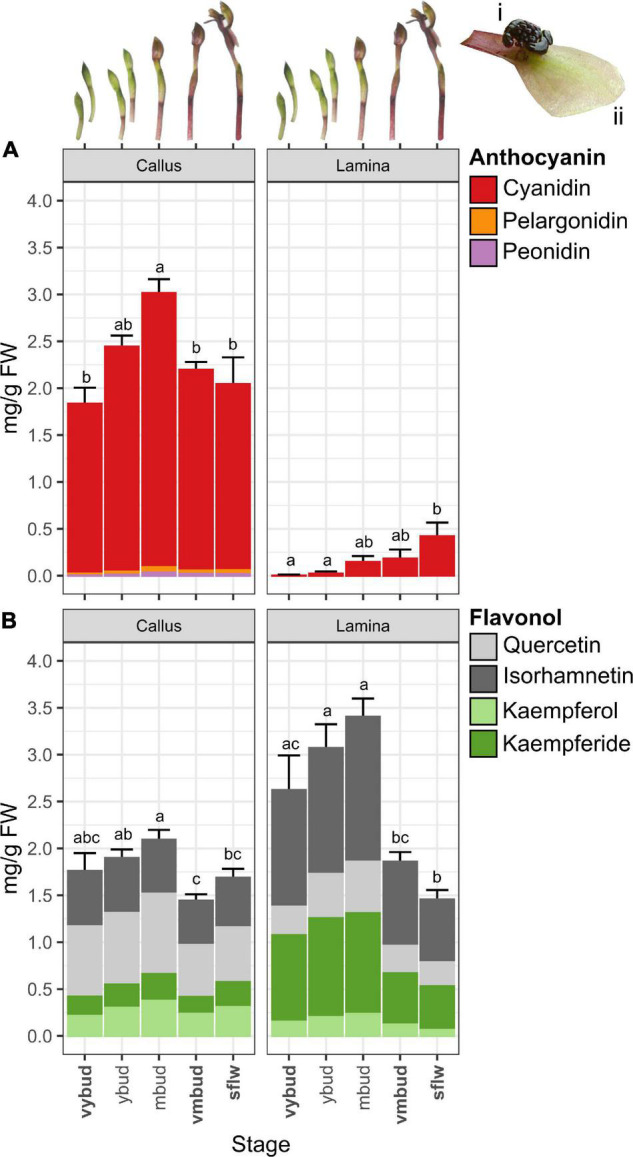
Changes in anthocyanins and flavonol glycoside co-pigments of *Chiloglottis trapeziformis* flowers. Bar graphs depict the total **(A)** anthocyanin and **(B)** flavonol glycoside content (average ± s.e.) in the callus and labellum lamina (see insets i and ii) of *Chiloglottis trapeziformis* flowers at different developmental stages (i.e., very young bud, *vyb*; young bud, *yb*; mature buds, *mb*; very mature bud, *vmb*; and naturally opened flowers in the field, *sflw*). Bold indicates developmental stages with corresponding transcriptome profiled. Labels not connected by the same letter are significantly different at *P* < 0.05 based on ANOVA and Tukey HSD test (see Supplementary Figure 7 for further information).

The classifications of bud and flower stages follows Amarasinghe et al. (2015). Briefly, the *vyb* is characterized by a small and very tightly closed green bud. However, it is noteworthy that dissection of the buds reveals that the callus is already very dark in color at the *vyb* stage. The *vmb* stage is characterized by larger green buds that are about to open, with sepals and petals beginning to separate. By this stage the callus is black, and remains so for the duration of flowering. At the onset of flower opening, the lamina is green. However, within 2 to 3 days of exposure to sunlight, the lamina becomes reddish-green in color. It was at this stage that the *sflw* was sampled (Figure 2 inset).

### Ultra High-Performance Liquid Chromatography Mass Spectrophotometric Analysis

For the targeted metabolite analysis, a total of 54 separate labellum samples were used, encompassing replication for all of the five developmental stages (i.e., *vyb*, *yb*, *mb*, *vmb*, *sflw*). Each sample consisted of either the dissected callus or lamina (labellum remains after the stalked callus was removed) from several individual flowers. Pre-weighed samples were homogenized and extracted in 400 μL of solvent composed of 70:30:1 methanol:water:acetic acid with agitation at 4°C for 24 h. Samples were centrifuged at 13,000 rpm for 10 min and the supernatant was filtered with 0.2 μm PTFE SINGLE StEP filter vials (Thomson) and analyzed using a Thermo Q Extractive Plus UPLC-Orbitrap Mass Spectrometer (Thermo Fisher Scientific, Waltham, MA, United States). Samples and standards (5 μL injection volumes) were separated chromatographically on an Agilent reversed-phase Zorbax Eclipse XDB-C18 column (2.1 × 50 mm, 1.8 μm particles) held at 40°C. The mobile phases used were water with 0.1% HPLC grade formic acid (solvent A), and methanol with 0.1% formic acid (solvent B). Samples and standards were eluted with a constant flow rate of 400 μL min^–1^, with a 25-min gradient program as follows: 0–1.5 min, 6% B; 1.5–2 min, 6–10% B; 2–14 min, 10–60% B; 14–15 min, 60–90% B; 15–19 min, 90% B; 19–20 min, 6% B; 20–25 min, 6% B. Eluted compounds were introduced to the MS via a HESI-II probe (Thermo Fisher Scientific, Waltham, MA, United States). Representative samples were also analyzed on a separate LC-MS instrument fitted with a diode array detector (DAD) operating at 520 nm and at 365 nm and using the same chromatographic parameters, to distinguish anthocyanins from flavonol glycosides.

For putative identification and relative quantification of anthocyanins and flavonols, the HESI was operated in the positive mode. Mass spectra were acquired using full MS and data-dependent MS/MS acquisition (DDA) modes at a scan range of 100 to 1500 m/z. Additionally, representative samples of both tissue types at various developmental stages were analyzed in negative ionization mode. In negative ionization mode, anthocyanins produce a distinctive [M-2H + H_2_O]^−^ ion in addition to the [M-2H]^−^ ion, while isomeric flavonol glycosides do not (Sun et al., 2012a). Thus, the presence of this ion is diagnostic of anthocyanins and can be used to distinguish them from isomeric flavonols with similar fragmentation patterns.

Data were acquired using Thermo Scientific XCALIBUR 4.0 and analyzed using Thermo Scientific FreeStyle software. Anthocyanins and flavonols were distinguished based on (i) accurate masses (4 decimal places) of molecular ions in full MS spectra in both ionization modes, (ii) comparisons of fragmentation patterns in the MS/MS spectra in both ionization modes with those available in online databases such as RIKEN tandem mass spectral database (Sawada et al., 2012) and MassBank of North America^1^, (iii) the presence or absence of the diagnostic [M-2H + H_2_O]^−^ ion in the negative ionization analyses, and (iv) absorbance spectra at 520 and 365 nm. Putative anthocyanins and flavonols were quantified by integration of the molecular ion peak in full MS spectra and calculated from linear calibration curves of cyanidin 3-O-glucoside chloride (Sigma-Aldrich) and 7-hydroxycoumarin (Sigma-Aldrich), respectively.

An analysis of variance (ANOVA) and *post hoc* Tukey–Kramer Honestly Significant Difference test were performed to evaluate the differences in total anthocyanin and flavonol glycoside content in the callus and labellum during flower development, following the confirmation of normality by a Shapiro-Wilk test. Statistical analyses were conducted using R^2^.

### *De novo* Transcriptome Assembly

RNA extraction, library construction, and RNA sequencing were performed as previously described (Wong et al., 2017a,^2018^). However, the assembly and downstream analysis of the *sflw* stage has not been previously reported. Therefore, paired-end reads from the callus and lamina tissues of *vyb*, *vmb* (Wong et al., 2017a,^2018^) and *sflw* (used for the first time in this study) stages were first pooled. Next, removal of the adaptor, sliding-window trimming, length (*l* = 40) and quality filtering, and base correction (*-c*) of the raw PE reads were performed with *fastp* v0.20.0 (Chen et al., 2018) using default settings unless otherwise specified. *De novo* transcriptome construction based on pooled tissue and developmental stages was performed using *Trinity* v2.11.0 (Haas et al., 2013) with default settings except the minimum contig length and *k*-mer size were set to 300 and 31, respectively. Protein-coding prediction was achieved using TransDecoder v5.5.0^3^. To maximize the prediction sensitivity, homology searches against the UniProt Reference Clusters (UniRef90) and protein families (Pfam) databases were performed using DIAMOND (Buchfink et al., 2014) and HMMER (Mistry et al., 2013), and incorporated into the TransDecoder pipeline. Resolution of assembly redundancy, protein-coding region prediction, and identification of accurate gene sets was made using the EvidentialGene tr2aacds4 pipeline (Gilbert, 2013) using default settings.

### Transcriptome Analysis of Anthocyanin and Flavonol Glycoside Pathway-Related Genes

Alignment of filtered PE reads toward the assembled transcriptome of *C. trapeziformis* were individually performed with bowtie2 (Langmead and Salzberg, 2012) using the local read alignment mode (*–local*). Read count matrices were obtained using FeatureCounts (Liao et al., 2014) with default parameters except for the *-B* (both ends must be aligned) and *-C* (exclude chimeric fragments) option enabled for each species. Transcripts having sufficiently large counts were retained for further downstream statistical analysis using edgeR with the *filterByExpr* option (Robinson et al., 2009). Differential expression (DE) analysis between groups/treatments of interests were performed using DESeq2 (Love et al., 2014). An absolute log_2_ fold change (| log2FC|) > 0.5 and a false discovery rate (FDR) threshold < 0.05 determines transcripts that are differentially expressed in each contrast. Transcript expression was expressed as Fragments Per Kilobase of transcript per Million mapped reads (FPKM). The assembled transcriptome was annotated with MapMan categories using Mercator (Lohse et al., 2014). Additional Pfam domains, putative homolog annotations, and transcripts sharing homology and functional domains to known and/or emerging flavonoid pathway components (Grotewold, 2006; Wessinger and Rausher, 2012; Tohge et al., 2017), were obtained via the TransDecoder’s prediction pipeline. Hierarchical clustering of expression and DE patterns of the callus and lamina across multiple developmental stages were performed using *hclust* and plotted using the *ggplot2* package in R (see text footnote 2). A summary of developmental stage- and tissue-specific transcriptome analysis of *Chiloglottis trapeziformis* flowers is described in Supplementary Figure 1 and corresponding gene expression dataset and differential expression result tables are available in Supplementary Datas 1–3. New sequence reads obtained for the *sflw* stage has been added to the existing BioProject accession PRJNA390683 and SRA study accession SRP1093281.

## Results

### The Callus and Labellum Contain a Diverse Array of Anthocyanin and Flavonol Glycosides

A total of five anthocyanins and 14 flavonol glycosides were detected in *Chiloglottis trapeziformis* labella by UHPLC-MS/MS and LC-DAD-MS analysis (Table 1 and Supplementary Figure 2). Cyanidin-based anthocyanins were the dominant pigment, with peonidin- and pelargonidin-based anthocyanins also detected. Kaempferol (K), Quercetin (Q), and their methylated derivatives (Kaempferide, Kde; Isorhamnetin, IR) were the dominant flavonol glycosides. No delphinidin-based anthocyanins, myricetin flavonol glycosides nor common acyl modifications aside from malonyl groups (e.g., acetylation, coumarylation, caffeoylation) were detected (Table 1).

**TABLE 1:** Putative anthocyanins and flavonols in floral extracts of *Chiloglottis trapeziformis* calli and labellum lamina tissues.

Putative anthocyanin/flavonol glycoside	Putative aglycone	Peak RT (min)	[M]+	[M]+ MSMS transitions (relative intensity)	[M-2H]-, [M-2H + H2O]-
**Anthocyanin**					
Cyanidin glucoside (CG)	Cyanidin	7.12	449.1076	**287.0547 (100),** 288.0580 (8.5), 71.7628 (0.9), 286.9883 (0.7), 69.7407 (0.3)	447.0931, 465.1034
Cyanidin malonylglucoside (CmG)	Cyanidin	8.93	535.1086	**287.0546 (100),** 288.0579 (7.4), 71.7628 (0.9), 286.9883 (0.7), 258.0482 (0.06)	533.0936, 551.1042
Pelargonidin glucoside (PelG)	Pelargonidin	7.54	433.1131	**271.0596 (100),** 272.0630 (10.3), 67.7641 (1.0), 270.9985 (0.6), 69.7845 (0.4)	431.0979, 449.1090
Peonidin glucoside (PeoG)	Peonidin	8.03	463.1233	**301.0702 (100),** 302.0735 (10.5), 75.2666 (0.9), 300.9990 (0.7), 286.0462 (0.4)	461.1088, 479.1197
Peonidin malonylglucoside (PeomG)	Peonidin	9.76	549.1234	**301.0701 (100),** 302.0734 (8.5), 75.2666 (0.9), 300.9991 (0.7), 286.0456 (0.4)	547.1096, 565.1200
**Flavonol glycoside**					
Quercetin diglucoside (QGG)	Quercetin	8.91	627.1541	**303.0492 (100),** 85.0287 (6.8), 304.0526 (5.9), 127.0388 (2.0), 97.0285 (1.8), **465.1016 (1.0)**	
Kaempferol diglucoside (KGG)	Kaempferol	8.95	611.1599	**287.0545 (100**), 85.0287 (10.4), 288.0579 (7.1), 97.0286 (2.8), 127.0388 (2.2), **449.1056 (2.0)**	
Quercetin malonyl diglucoside (QmGG)	Quercetin	9.56	713.1547	**303.0493 (100),** 127.0389 (8.7), 85.0287 (8.4), 304.0527 (8.2), 109.0285 (5.3), **465.1026 (2.4)**	
Quercetin glucoside (QG)	Quercetin	10.39	465.1024	**303.0494 (100),** 85.0287 (10.7), 304.0527 (9.5), 97.0286 (2.8), 127.0389 (2.5),	
Unknown Quercetin pentose (QP)	Quercetin	10.48	435.0925	**303.0496 (100),** 73.02881 (15.7), 304.0534 (10.1), 195.0285 (4.6), 57.0341 (4.8)	
Quercetin malonylglucoside (QmG)	Quercetin	10.83	551.1034	**303.0496 (100),** 127.0390 (11.7), 85.0288 (11.6), 109.0286 (9.1), 304.0529 (7.8)	
Kaempferol glucoside (KG)	Kaempferol	11.43	449.1076	**287.0546 (100),** 85.0288 (9.3), 288.0580 (6.9), 97.0286 (3.0), 127.0389 (2.5)	
Isorhamnetin diglucoside (IRGG)	Isorhamnetin	11.81	641.1708	**317.0651 (100),** 318.0686 (6.2), 85.0287 (4.4), 97.0286 (1.7), 79.2659 (1.6), **479.1159 (1.2)**	
Kaempferide diglucoside (KdeGG)	Kaempferide	11.84	625.1761	**301.0701 (100),** 302.0735 (7.6), 85.0287 (7.2), **463.1230 (2.6),** 97.0285 (2.0)	
Kaempferol malonylglucoside (KmG)	Kaempferol	11.86	535.1075	**287.0546 (100),** 127.0390 (11.0), 85.0287 (9.5), 109.0285 (6.9), 159.0285 (4.5)	
Isorhamnetin malonyl diglucoside (IRmGG)	Isorhamnetin	12.21	727.1708	**317.0649 (100**), 318.0683 (9.3), 85.0287 (6.6), 127.0389 (6.0), 109.0284 (4.1), **479.1175 (2.8)**	
Kaempferide malonyl diglucoside (KdemGG)	Kaempferide	12.32	711.1749	**301.0704 (100**), 85.0288 (8.2), 302.0736 (7.7), 127.0390 (7.6), 109.0285 (5.4), **463.1222 (4.6)**	
Isorhamnetin glucoside (IRG)	Isorhamnetin	13.89	479.1182	**317.0650 (100)**, 318.0684 (10.2), 79.2653 (0.9), 316.9880 (0.8), 85.0287 (0.7)	
Isorhamnetin malonylglucoside (IRmG)	Isorhamnetin	14.34	565.1182	**317.0649 (100)**, 127.0389 (10.4), 85.0287 (8.8), 318.0684 (7.6), 109.0285 (6.7)	

*The positions of sugar and acyl group attachment to the anthocyanidins or flavonol aglycones were not determined, and glucosides cannot be distinguished from isomeric galactosides using our methodology. Especially informative MSMS transitions are listed in bold.*

### Candidate Anthocyanin and Flavonol Glycoside Pathway-Related Genes

Our transcriptome analysis revealed a set of candidate gene homologous to well-known anthocyanin and flavonol glycoside pathway genes (Grotewold, 2006; Tanaka et al., 2008; Davies et al., 2012). This included three chalcone synthase (*CtrCHS1–3*), one chalcone isomerase (*CtrCHI*), two flavone 3-hydroxylase (*CtrF3H1–2*), and four flavonoid 3’-hydroxylase (*CtrF3’H1–4*) encoding enzymes involved in the early biosynthetic steps of general flavonoid biosynthesis. Notably, F3’H catalyzes hydroxylation at the 3’-position of naringenin and dihydrokaempferol to yield eriodictyol and dihydroquercetin, and also converts kaempferol to quercetin (Tohge et al., 2017). Three dedicated anthocyanidin biosynthesis genes (one *CtrDFR1* and two leucoanthocyanidin dioxygenase syn. anthocyanidin synthase) (*CtrLDOX1–2*) and two flavonol glycoside biosynthesis genes (e.g., flavonol synthase, *CtrFLS1–2*) were also identified. The discovery of the candidate genes downstream of F3’H is in accord with the anthocyanins (two cyanidin and two peonidin anthocyanins) and flavonol glycosides (five Q and four IR flavonol glycosides) we detected. One pelargonidin anthocyanin, three K and three Kde flavonol glycosides which do not depend on F3’H activity were also found (Figure 2 and Table 1). Many of these genes also fall within the specific BIN9.2.2 category of secondary metabolism.phenolics.flavonoid biosynthesis (e.g., *CtrCHS* in BIN9.2.2.1, *CtrCHI* in BIN9.2.2.2, *CtrF3H* in BIN9.2.2.4, and *CtrDFR* and *CtrLDOX* in BIN9.2.2.9) or were reciprocal best BLAST hits of several plant CYP75B (i.e., *CtrF3’H*) enzymes (Supplementary Data 1).

Unlike the conserved early steps in the pathway, the downstream modifications of flavonoids with various glycosyl, methyl, and/or acyl groups are achieved by highly diverse and often family, genera-, and/or even species-specific sets of genes (Tanaka et al., 2008). Nonetheless, typically one or more flavonoid glycosyltransferases, methyltransferases, or acyltransferases catalyze these reactions. Three glycosyltransferase homologs potentially relevant to flavonoid metabolism were identified: two transcripts encoding Arabidopsis UDP-glucose:flavonoid 3-O-glucosyltransferase homolog (*CtrUGT78D2a/b*) potentially involved in the glycosylation of anthocyanidins and flavonols at the 3-position (Lim et al., 2004; Tohge et al., 2005), and one encoding an Arabidopsis UGT71B1 (Lim et al., 2004) homolog with specific flavonol 3-O-glucosyltransferase activity (*CtrUGT71B1*).

*O*-methyltransferases with both broad and narrow flavonoid substrate specificities have been identified in many plants. For example, the grape anthocyanin *O*-methyltransferase VviAOMT that catalyzes the methylation of anthocyanins is also active on flavonol glycosides compounds (Hugueney et al., 2009; Lücker et al., 2010; Provenzano et al., 2014). Here, four transcripts sharing homology toward VviAOMT (*CtrCOMT1–4*) were identified. Many plant flavonoid malonyltransferases characterized to date can catalyze the malonylation of anthocyanins but also flavonol glycosides (Bontpart et al., 2015) and one such enzyme is dahlia anthocyanin 3-glucoside malonyltransferase, Dv3MaT (Suzuki et al., 2002). Four *C. trapeziformis* homologs of Dv3MaT were identified (*Ctr3MAT1–4*).

Subcellular flavonoid transport homologs such as Arabidopsis glutathione-S-transferase, Transparent Testa 19 (TT19) involved in the transport of cyanidin anthocyanins from the cytosol to the tonoplast (Sun et al., 2012b), and various plant ABC and MATE transporters that also facilitate the vacuolar transport of anthocyanins and flavonoids such as grape ABCC1, *A. thaliana* ABCC2, and grape anthoMATEs (Gomez et al., 2009; Francisco et al., 2013; Behrens et al., 2019) were also identified (e.g., *CtrTT19a/b*, *CtrABCC1*, *CtrAM1*, and *CtrABCC2a/b*). These include transcripts encoding homologs of petunia H^+^ P-ATPase PH5 and Na^+/^H^+^ and K^+^/H^+^ antiporter NHX1 (Faraco et al., 2014) and Japanese morning glory NHX1 (Fukada-Tanaka et al., 2000) involved in the regulation of vacuolar pH were also identified (*CtrPH5* and *CtrNHX1*). Finally, a homolog to the gene encoding CHIL that binds to CHS to serve as a rectifier, thus, facilitating the flux of phenylpropanoid pathway precursors to the flavonoid pathway (Waki et al., 2020), was also found (*CtrCHIL*).

### Developmental- and Tissue-Specific Expression of Anthocyanin and Flavonol Glycoside Pathway-Related Genes

In this study, we considered four pairwise developmental stage comparisons between the *vyb*, *vmb* (devC1/L1) and *sflw* (devC2/L2) stages across the callus and lamina: Three tissue-specific comparisons between callus and lamina across the respective *vyb* (ts1), *vmb* (ts2), and *sflw* (ts3) developmental stages corresponding with the samples profiled for anthocyanin and flavonol glycosides. First, we clustered candidate anthocyanin and flavonol glycoside pathway-related genes based on their expression abundance (Supplementary Figure 3). Four clusters (cluster A–D) of shared expression intensities were identified. Most notably, *CtrCHS1*, *CtrF3H1/2*, and *CtrLDOX1* were highly expressed (FPKM > 100) especially in the callus and/or lamina of *vyb* (Cluster D) while lowly expressed (FPKM < 1) transcripts such as *CtrF3pH2/3*, *CtrMAT4*, and *CtrCHS2/3* were observed in Cluster C. Furthermore, hierarchical clustering based on the patterns of differential expression revealed clusters containing genes that were differentially expressed between callus and lamina in both specific and multiple developmental stages (Supplementary Figure 4). For example, several groups of genes were coordinately downregulated to varying degrees (i.e., cluster 3–5) during the transition from *vyb* to *vmb* and *sflw* stage regardless of tissue type. In cluster 5, there were many genes encoding enzymes involved in the formation of early anthocyanin and flavonol precursors such as chalcones, flavanones, and dihydroflavonols. These include two *CHS* (*CtrCHS1/3*), one *CHI* (*CtrCHI1*), two *F3H* (*CtrF3H1/2*), and two *F3’H* (*CtrF3pH1/4*) genes. Conversely, cluster 3 contained mostly downstream flavonoid modification genes and displayed stronger and more consistent developmental stage downregulation in both tissues compared to other clusters. Across several clusters in both tissue types over flower development, coordinated regulation was not limited to the upstream biosynthetic pathway but also included downstream modification and/or transport genes. For example, *CtrAM1/2*, *CtrPH5*, and two flavonoid-related glycosyltransferase homologs *CtrUGT71B1* and *CtrUGT78D2b* were present in cluster 5, *CtrTT19b* in cluster 2, and *CtrCOMT1/4* in cluster 4. However, cluster 1 and 3 mostly contained flavonoid modification or transport genes, while the majority of transcripts in cluster 1 encoded downstream transport genes and displayed developmental stage upregulation in both the callus and lamina.

### The Chemical Basis and Patterns of Gene Expression in the Black Callus

The total anthocyanin content was substantially higher in callus tissue compared to the lamina across all developmental stages (Figure 2A). For example, at the *vyb* stage, total anthocyanin quantities were up to 1300 times higher in the callus than in the lamina. Even after the late peak of anthocyanin production in the *sflw* stage, the callus anthocyanin content remained 5 times greater than in the lamina. Across developmental stages, total anthocyanin content of the callus averaged 1.8 mg g^–1^ FW in *vyb* stage, before peaking in *mb* (3.0 mg g^–1^ FW) and then decreasing in *vmb* and *sflw* to comparable levels observed in *vyb* (2.1–2.2 mg g^–1^ FW). The majority of anthocyanins were cyanidin-based (96–97%) regardless of developmental stage, with relatively low amounts (0.02–0.06 mg g^–1^ FW) of peonidin- and pelargonidin-based anthocyanins (Supplementary Figure 5). Across the different developmental stages, the pattern of accumulation for individual anthocyanins were largely consistent with total anthocyanin content (Supplementary Figure 5).

Four main types of flavonol glycosides were found in the callus, with the proportions of quercetin (Q)-, isorhamnetin- (IR), kaempferol- (K), and kaempferide- (Kde) based flavonol glycosides ranging between 36–44%, 26–32%, 12–18%, and 12–16%, respectively (Supplementary Figure 6). Compared to the anthocyanins, less variation in total flavonol glycoside content was found across the different bud (1.4–2.1 mg g^–1^ FW) and flower (1.7 mg g^–1^ FW) stages (Figure 2B). Major flavonol glycosides such as QG and IRG were often most abundant in early developmental stages (i.e., pre *mb* before decreasing slightly in *vmb* and *sflw*) while others such as KG, K(mal)G, and KdeGG showed subtle increments from *vyb* to *mb* before decreasing in *sflw* (Supplementary Figure 6). However, the ratios of total anthocyanins to flavonol glycosides in the callus were broadly similar across developmental stages (approximately 1.5 to 1).

**FIGURE 3:**
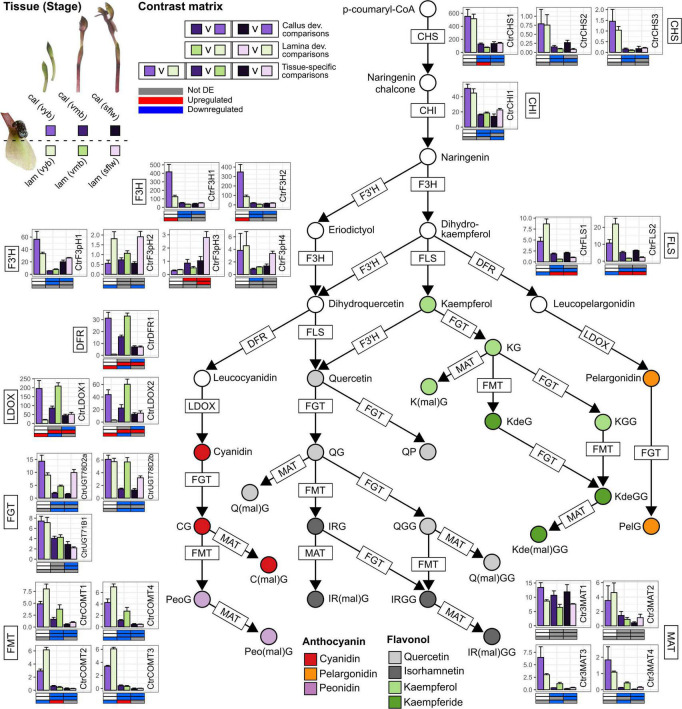
Anthocyanin and flavonol metabolism in the callus and labellum tissues of *Chiloglottis trapeziformis* during flower development. Bars depict the average (±s.e.) normalized expression values (FPKM) of anthocyanin and flavonol biosynthesis and modification pathway genes in the calli and labellum lamina tissues of very young buds (*vyb*), very mature buds (*vmb*), and mature sunflowers (*sflw*). Colored circles indicate putative anthocyanins and flavonols identified in *Chiloglottis trapeziformis* callus (cal) and labellum lamina (lam) tissues (Table 1). Various shades of purple and green depict the relevant color callus and labellum lamina tissues at various flower developmental stages. The contrast matrix indicates the differential expression outcomes based on comparisons in Supplementary Figure 1. Red, blue, and gray indicate significant upregulation (FDR < 0.05, log_2_FC > 0.5), downregulation (FDR < 0.05, log_2_FC < –0.5), and no significant differential expression in selected comparisons, respectively. CHS, Chalcone synthase; CHI, Chalcone isomerase; F3H, Flavanone 3-hydroxylase; F3′H, Flavonoid 3′-hydroxylase; F3′5′H, Flavonoid 3′5′-hydroxylase; DFR, Dihydroflavonol 4-reductase; LDOX, Leucoanthocyanidin dioxygenase; FLS, Flavonol synthase; FGT, Flavonoid/anthocyanin glucosyltransferase; FMT, Flavonoid/anthocyanin O-methyltransferase; MAT, Flavonoid/anthocyanin malonyltransferase.

One key pattern of gene expression in the callus, especially at the *vyb* stage, was that genes related to anthocyanin and/or flavonol glycoside biosynthesis (e.g., *CtrF3H1/2*, *CtrDFR1*, *CtrLDOX1/2*), transport (e.g., *CtrAM1/*2, *CtrTT19b*), and vacuolar acidification (*CtrPH5*)-related pathways showed significantly higher gene expression when compared to the lamina. Most notably, *CtrDFR1* and *CtrLDOX1/2* transcripts peaked in the callus of *vyb* and were 10–35-fold higher compared to the lamina (Figure 3 and Supplementary Figure 4). Despite evidence for coordinated downregulation, and hence lower gene expression levels for many of these genes from *vmb* onward, some transcripts such as *CtrCHS1*, *CtrFLS1/2*, *CtrMAT1*, *CtrCOMT2/3*, *CtrNHX1*, *CtrAM1/2*, and *CtrABCC2a/b* continued to exhibit higher gene expression levels in the callus compared to the lamina in one or more stages post *vyb*.

### The Chemical Basis and Patterns of Gene Expression in the Green Lamina

While the total anthocyanin content was much lower in the lamina than the callus, trace levels were nonetheless detected from the *vyb* stage (0.014 mg g^–1^ FW) with a steady increase to appreciable amounts at the *sflw* (0.42 mg g^–1^ FW) stage. These patterns match the observed transition from green buds to the reddish green mature flower. Like the callus, cyanidin-based anthocyanins were the major anthocyanin constituent of the lamina regardless of developmental stages (99–100%), with very low amounts of peonidin- and pelargonidin-based anthocyanins. Accordingly, CG and C(mal)G content increased steadily from *vyb* to *sflw* in the labellum. Interestingly, total flavonol glycoside content in the lamina closely mirrored the profile of total anthocyanins in the callus (Figure 1B). The average total flavonol glycoside content increased from 2.6 to 3.4 mg g^–1^ FW in the *vyb* and *mb*, before decreasing sharply (1.5 mg g^–1^ FW) in *sflw*. This decreasing pattern is concomitant with the sharp rise in total anthocyanins from *vyb* to *sflw*.

When compared with the callus, there was substantial variation in the ratio of total flavonol glycosides to anthocyanins across developmental stages of the lamina with the top two-most extremes observed in *vyb* (1800:1) and *sflw* (3.5:1). Notably, the total (and most individual) flavonol glycoside content in the lamina of *sflw* was the lowest of all developmental stages, directly contrasting with the total anthocyanin content which was greatest in this stage (Figure 2). Regardless of developmental stage, isorhamnetin (43–46%) and kaempferide (29–35%) flavonol glycosides were more prevalent in the lamina, while quercetin- (12–19%) and kaempferol-based flavonol glycosides (5–7%) were less prevalent relative to the callus (Supplementary Figure 6). Unlike the callus, in the lamina most major flavonol glycosides either displayed a marked increase in content (e.g., QG, IRGG) or remained constant [e.g., IRG, KdeGG, Kde(mal)GG] during the transition from *vyb* to *mb*, followed by a large decrease in the mature stages (Supplementary Figure 6).

Coordinated regulation of *CtrFLS1/2* and *CtrCOMT2/3* transcripts (cluster 4) was a striking feature of the lamina. Four transcripts were significantly upregulated in the lamina compared to callus of *vyb*, however, at later stages of *vmb* and *sflw*, higher callus expression was commonly observed. Coordinated downregulation during the transition from *vyb* to *vmb* and *sflw* stages was also common for many anthocyanin and flavonol glycoside pathway genes in the lamina (i.e., cluster 3–5). Another striking feature of the lamina was a strong (but lagging the callus) developmental upregulation of genes encoding *CtrDFR1*, *CtrLDOX1/2*, *CtrAM1/*2, and *CtrTT19b* (cluster 2). For example, *CtrDFR1* was consistently upregulated by 12- to 55-fold in *vmb* and *sflw* compared to *vyb*. Similarly, *CtrLDOX1/2* were upregulated by four- to 30-fold in *vmb* and flowers compared to *vyb*. Interestingly, these transcripts were only upregulated in the lamina compared to the callus at the *vmb* stage. Other notable transcripts include *CtrUGT78D2a/b*, *CtrMAT3/4*, *CtrCOMT2/3*, and *CtrPH5*, which were more highly expressed in the labellum than the callus in one or more developmental stages post *vyb* (Figure 3 and Supplementary Figure 4).

## Discussion

### Overview

The goal of this study was to combine targeted metabolite profiling and transcriptomic analysis to investigate the chemical composition and patterns of tissue- and developmental-specific color pathway gene expression in the labellum of *Chiloglottis trapeziformis*. In this sexually deceptive orchid (as in most others), the labellum is the center of the orchid-pollinator interaction, functioning as the olfactory, visual, and tactile mimic of the female of the pollinating species (de Jager and Peakall, 2016, 2019). Furthermore, the color contrast between the dark black 3D callus structure mimicking the female (Figure 1A) and the remainder of the reddish green labellum lamina is predicted to aid detectability to the male pollinator. In the discussion that follows, we explore in more detail hypotheses for the molecular basis of the contrasting labellum colors, and consider evolutionary implications and future research directions.

### The Diversity of Anthocyanin and Flavonol Co-pigments in *Chiloglottis trapeziformis*

Cyanidin-based anthocyanins are widespread floral pigments across the flowering plants, and are also known from many orchid species (Iwashina, 2015). However, as one of the largest plant families, it is not surprising that anthocyanins have only been investigated in a fraction of the known genera and species of orchids. Pigment chemistry and biosynthesis has been most well studied in orchid genera of horticultural interest, such *Phalaenopsis* (Hsu et al., 2015; Liang et al., 2020), *Vanda* (Khunmuang et al., 2019), *Cattleya* (Li et al., 2020), and *Cymbidium* (Wang et al., 2014) but there have also been several studies of wild species which span a diversity of orchids with both rewarding and deceptive pollination strategies (George et al., 1973; Strack et al., 1989; Fossen and Øvstedal, 2003; Vignolini et al., 2012; Kellenberger et al., 2019; Zhang et al., 2020). Notably, cyanidin-based anthocyanins are major anthocyanin pigments of flowers in the European sexually deceptive genus *Ophrys* (Strack et al., 1989; Vignolini et al., 2012). Unlike anthocyanins, the constituent flavonol glycosides (or any other flavonoid classes) remain poorly understood across the Orchidaceae.

Despite the exceptional diversity (>1850 species) of the Australian orchid flora (Jones, 2021; Peakall et al., 2021), to the best of our knowledge only one previous study of floral anthocyanins has been published. This study, dating back to the 1970s, reported a qualitative survey of floral anthocyanins across some brightly colored and non-sexually deceptive species. The finding indicated the presence of cyanidin-, delphinidin-, petunidin- and malvidin-based anthocyanins (George et al., 1973). The present study is thus the first to investigate in detail the chemical and molecular basis of flower color in an Australian orchid. We have confirmed that cyanidin-based anthocyanins are the major pigment constituents of the labellum of *Chiloglottis trapeziformis* flowers. The labellum lamina also accumulates other mono- and di-hydroxylated anthocyanins and flavonol glycosides, but no trihydroxylated forms were detected. Additionally, among the many possible acyl modifications of the anthocyanin and flavonol glycosides, only the malonylated form was detected (Table 1). The composition of flavonol glycosides was more complex than the anthocyanin profile in both tissue types. In the callus, isorhamnetin- and quercetin-based compounds were equally dominant while the lamina accumulated more isorhamnetin- and kaempferide-based flavonol glycosides (Figure 2B and Supplementary Figures 5, 6).

### Patterns of Anthocyanin and Flavonol Glycoside Levels in *Chiloglottis trapeziformis*

Dissection of the tightly closed green bud at the *vyb* stage shows that the callus is already darkly colored. By the *mb* stage the callus appears “black” in color, and it remains so for the lifetime of the flower (Amarasinghe et al., 2015). On the other hand, the color of the lamina changes from uniform green at *vyb* to greenish red at *vmb*, but only reaches its maximum reddish green color in flowers that have been open in sunlight for several days (Amarasinghe et al., 2015). Consistent with these observations, peak anthocyanin accumulation was observed in the *vmb* stage of the callus, compared with the final *sflw* stage of the lamina (Figure 2A). Furthermore, the lower levels of lamina anthocyanins are spread thinly across the larger surface area, compared with the smaller callus (Figure 1A). Reflectance measurements, projected into hymenopteran vision space, indicate that the dark achromatic callus should be readily distinguished by the pollinator from the contrasting lamina, even at full flower maturity (de Jager and Peakall, 2016).

By contrast with the anthocyanins, total flavonol glycoside levels were broadly similar across callus and lamina tissues and seemingly inversely correlated with the total anthocyanin levels. Nonetheless, some different patterns of composition and changes with development stage were evident (Figure 2B and Supplementary Figure 6). Critically, when compared to the lamina, lower levels of the methylated forms of Quercetin (Isorhamnetin) and Kaempferol (Kaempferide) were found in the callus (Figure 2B).

### Patterns of Gene Expression in the Flower Color Pathway Genes of *Chiloglottis trapeziformis*

Figure 3 shows an overlay of the patterns of gene expression in the *C. trapeziformis* labellum partitioned by tissue type and development stage onto the general biosynthetic pathway for anthocyanin and flavonoid production in plants. The gene expression patterns in combination with the outcomes of the hierarchical clustering analysis (Supplementary Figures 3, 4) and knowledge of the anthocyanin and flavonol glycoside levels (Figure 2), provide insights into the probable genetic basis of the contrasting labellum colors of *C. trapeziformis*.

The two early pathway genes, *CtrCHS1-3* and *CtrF3H1-2*, were equally highly expressed in both callus and lamina tissues of the *vyb* stage, indicating an efficient supply of precursors is available for both anthocyanin and flavonol glycosides biosynthesis in either tissue. Whereas, the dedicated anthocyanin pathway genes associated with the production of anthocyanins (cyanidin, peonidin, and pelargonidin), *CtrDFR1*, *CtrLDOX1-2* were highly expressed in the *vyb* stage of the callus, but not the lamina. Conversely, the *CtrFLS1-2* associated with the production of the flavanol glycosides, were significantly downregulated in the callus tissue. The methyltransferase, FMT (*CtrCOMT1-4*) genes mirrored the *FLS* gene expression patterns, consistent with the observed increasing accumulation of the methylated flavonol glycosides in the lamina from *vyb* to *mb* stages (Figure 3 and Supplementary Figure 6).

By the *vmb* stage, expression levels of the precursor supply genes, CHS and CHI, were significantly downregulated relative to the *vyb* stage in both tissue types, and remained low across the remaining developmental stages. In the callus, the intermediate anthocyanin pathway genes (F3H, F’3H, DFR and LDOX), generally showed a similar pattern. However, across the remaining developmental stages, there was a strong lag in the peak expression of *CtrDFR1* and *CtrLDOX1-2* between the lamina and callus. This suggests that the combination of upregulation of the anthocyanin genes in the lamina, coupled with a diminished precursor supply is likely linked to the increasing but much lower levels of anthocyanin production in the lamina with development stage.

By the *sflw* stage, the formerly green color of the *vmb* stage, and the freshly opened flower was reddish-green in color. Consistent with this observation, anthocyanin levels in the lamina, while much lower than in the callus, peaked at the *sflw* stage (Figure 2A). Subtle gene expression shifts appear to be consistent with these flower color changes. For example, expression of CHS (*CtrCHS1*) and CHI (*CtrCHI1*) increased in both callus and lamina relative to the *vmb* stage. More importantly, callus expression was significantly upregulated relative to the lamina. Thus, it seems likely that precursor supply, while remaining diminished relative to the *vyb* stage, should increase somewhat in this final developmental stage (Figure 3).

### Patterns of Gene Expression in Genes Potentially Modifying the Flower Color of *Chiloglottis trapeziformis*

Beyond the direct anthocyanin and flavonol metabolism genes (Figure 3), other genes such as those involved in modification, transport, and sequestration, as well as genes associated with chlorophyll production may influence the flower color of *C. trapeziformis*. Notably, some genes involved in the modification of anthocyanins and flavonol glycosides showed uniform levels of gene expression across tissue types and developmental stages. This included the flavonoid glucosyltransferases, FGT (*CtrUGT78D2a/b* and *CtrUGT71B1*) and malonyltransferases, MAT (*Ctr3MAT1-4*) (Figure 3). Thus, it is likely that these downstream modification steps are not rate-limiting during the active accumulation of peak anthocyanins in the callus and the flavonols in the lamina (*vyb*-*mb* stages), nor in the final stage of the lamina becoming reddish green (*sflw*). We further predict that one or more of the MAT transcripts (e.g., *Ctr3MAT1-2*) are involved in the production of the malonylated anthocyanins (such as cyanidin malonylglucoside). Such derivatives were abundant (ca. 31–33% of total anthocyanins) in both the callus and labellum (Figure 2A and Supplementary Figure 5), and their accumulation profiles closely mirrored their non-malonylated anthocyanin precursors. The high abundance of malonylated anthocyanin derivatives in the callus, may be relevant to the preservation of its distinct dark color across developmental stages. Processes downstream of anthocyanin biosynthesis, such as the acylation of anthocyanins (e.g., malonylation) can have important consequences for floral color. For example, acylation has many functional roles including the protection of glycosides from enzymatic degradation, anthocyanin structure stabilization, and assistance in vacuolar uptake and sequestration (Bontpart et al., 2015). Importantly, these various functions may all serve to aid the stabilization of pigments in flowers (Suzuki et al., 2002; Luo et al., 2007).

Among the predicted transporter gene homologs, TT19 (*CtrTT19b*) showed a pattern of gene expression which mirrored *DFR* and *LDOX* genes (cluster 2, in Supplementary Figure 4). For example, at the *vyb* stage there was high and differential expression in the callus relative to the lamina, with this pattern reversing at the *vmb* stage. Expression levels in the callus plateaued at the *vmb* stage, while they peaked a second time in the lamina at the *sflw* stage. Thus, high expression levels at this gene appear to correspond with the respective starting points of anthocyanin accumulation and color development which started first in the callus with a peak by *mb*, but lagged in the lamina peaking at *sflw* (Figure 2A). In peach, expression of a glutathione S-transferase gene, *PpGST1* strongly correlates with anthocyanin accumulation in fruit tissues. Accordingly, transient overexpression of *PpGST1* increased the anthocyanin content in the fruit of yellow-fleshed nectarine while silencing of *PpGST1* in blood-fleshed (anthocyanin-rich) peach decreased anthocyanin accumulation resulting in white flesh (Zhao et al., 2020). Interestingly, *PpGST1* is also a locus containing functional and non-functional alleles that co-segregated with white and non-white (i.e., pink and red) flowers (Lu et al., 2021). In the purple-flowered cyclamen, expression of an anthocyanin-related GST, *CkmGST3* and upstream core biosynthetic genes such as *CkmF3’5’H* and *CkmDFR2* were tightly co-regulated during flower development. These transcripts were strongly expressed in paler pigmented petals and correlated with the sharp rise in anthocyanin accumulation compared to fully pigmented purple petal (Kitamura et al., 2012).

The expression patterns of a homolog to a gene implicated in the regulation of vacuolar pH, PH5 (*CtrPH5*), also showed some similarity to those of DFR, LDOX and TT19 genes (*CtrDFR1*, *CtrLDOX1/2*, and *CtrTT19b*) (Supplementary Figures 3, 4). For example, peak expression occurred in the callus at the *vyb* stage with significant downregulation as the flower developed. By contrast, in the lamina expression peaked in the *vmb* stage. Interestingly, at a homolog of NHX1 (*CtrNHX1*), gene expression levels were high in both tissue types at *vyb*, and tended to remain high in the callus across developmental stages. However, in the lamina, gene expression levels were differentially downregulated relative to the callus. These findings may indicate that vacuolar pH is lowest in the *vyb* stage of the callus, and in the *vmb* stage of the lamina. Furthermore, we predict that vacuolar acidification in the callus during active anthocyanin accumulation could play a synergistic role in conferring the strong “black” color of the callus. Different levels of vacuolar acidification are known to cause color shifts in flowers. For example, a less acidic vacuolar pH is often associated with blue colored flowers (e.g., *Petunia*, *Ipomea*, *Hydrangea*, and *Phalaenopsis* spp.) that otherwise share similar anthocyanin composition to other non-blue colored flowers (Fukada-Tanaka et al., 2000; Yoshida et al., 2003; Verweij et al., 2008; Liang et al., 2020). Presently, genes implicated in the regulation of vacuolar pH with relevance to flower color have only been established for a few species (i.e., Petunia and Japanese morning glory). Notably, the loss-of-function mutation of petunia *PH5* or the downregulation of *PH5* using RNA interference reduces vacuolar acidification in petals, resulting in “blue-violet” flower color in mutants and transformants compared to “red-violet” wild-type flowers (Faraco et al., 2014). Conversely, a mutation of morning glory *NHX1* impairs vacuolar alkalization, resulting in purple petals in fully bloomed flowers instead of bright blue colors in the wild-type (Fukada-Tanaka et al., 2000).

Chlorophyll is often present in petals during early developmental stages but usually decreases to trace levels in the typical brightly colored flowers of most plants. This loss of green coloration as the flower develops is considered crucial for making flowers more visible to animal pollinators against the green leafy background. However, examples of green colored flowers are nonetheless widespread across the flowering plants. Dull green and red flowers are particularly frequent among sexually deceptive plant species. These include hundreds of examples within the Australian genera *Caladenia*, *Chiloglottis*, *Cryptostylis*, *Drakaea* and *Pterostylis*, among others; tens of European *Ophrys* species, and several sexually deceptive species from South America (Bohman et al., 2016; Peakall et al., 2020).

Despite the widespread occurrence of green colored flowers and a thorough understanding of photosynthesis- and chlorophyll-related pathways (Hörtensteiner, 2006; Tanaka and Tanaka, 2006), little is known about how green colors occur in mature flowers. To date, the best clues are offered in carnations. Insights from carnation (*Dianthus caryophyllus* L.) floral transcriptomes reveal that expression of photosynthesis and chlorophyll biosynthesis genes are tightly associated with chlorophyll content, and thus coloration of green- and white-flowered cultivars (Ohmiya et al., 2014, 2017). Although the types and quantities of chlorophyll were not measured in this study, we predict that retention of chlorophyll pigments is pivotal for the green coloration of *Chiloglottis trapeziformis* flowers. When compared to the callus, the lamina was characterized by lower levels of anthocyanins and higher levels of colorless flavonol glycosides. Furthermore, in the lamina, consistently higher levels of gene expression were detected at photosynthesis- and chlorophyll-related genes across all developmental stages, indicative of enhanced chlorophyll biosynthesis in the lamina (Supplementary Figure 1 and Supplementary Data 1). Thus, the combination of low anthocyanins, high flavonol glycosides and sustained chlorophyll production are likely to be the basis of the predominant green coloration of the lamina from bud to flower opening.

### Transcriptional Control of Color Pathways in the Flower

**FIGURE 4:**
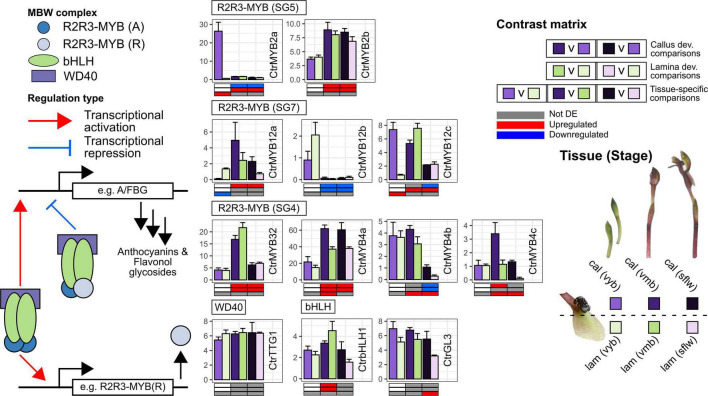
Transcriptional regulation of anthocyanin and flavonol glycoside pathway genes in the callus and labellum tissues of *Chiloglottis trapeziformis* during flower development. Bars depict the average (±s.e.) normalized expression values (FPKM) of putative transcriptional regulatory genes encoding relevant R2R3-MYB, basic-helix-loop-helix (bHLH), and WD40 repeat (WDR) proteins in the calli and labellum lamina tissues of very young buds (*vyb*), very mature buds (*vmb*), and mature sunflowers (*sflw*). A/FBG indicate shared flavonoid as well as anthocyanin/flavonol glycoside biosynthetic pathway genes. Various shades of purple and green depict the relevant color callus and labellum lamina tissues at various flower developmental stages. The contrast matrix indicates the differential expression outcomes based on comparisons in Supplementary Figure 1. Red, blue, and gray indicate significant upregulation (FDR < 0.05, log_2_FC > 0.5), downregulation (FDR < 0.05, log_2_FC < –0.5), and no significant differential expression in selected comparisons, respectively.

A detailed investigation of the transcriptional control of developmental and tissue-specific of pigmentation is beyond the scope of this study. Nonetheless, we highlight nine R2R3-MYB TF, two bHLH, and one WDR gene(s) potentially implicated in anthocyanin pigmentation in the flower of *Chiloglottis trapeziformis* (Figure 4 and Supplementary Data 4). The striking similarity in differential expression profile between *CtrMYB2a* and *CtrF3H1/2*, *CtrDFR1* and *CtrLDOX1/2* indicate that some shared and dedicated anthocyanin pathway genes are regulatory targets of *CtrMYB2a*. *CtrMYB2a*/*b* belongs to subgroup 5 of R2R3-MYB TFs that contains several known orchid tissue-specific floral pigmentation regulators (Chiou and Yeh, 2008; Hsu et al., 2015; Li et al., 2020). For example, the *CtrMYB2a* homolog in *Phalaenopsis*, *PeMYB2* is correlated with anthocyanin abundance in the petals and sepals of several cultivars and is highly expressed in full-red pigmented flowers. Transient overexpression of *PeMYB2* in white-flowered *P. aphrodite* ssp. *formosana* induced the expression of endogenous flavonoid and anthocyanin biosynthesis genes (e.g., *F3H*, *F3’H*, *DFR*, or *ANS* genes) and the development of reddish-pink pigmentation (Hsu et al., 2015).

Unlike orchid subgroup 5 R2R3-MYBs, the role of other subgroups (e.g., 6, 7, 20, and 4) in the regulation of flavonoid metabolism and pigmentation is unknown in the Orchidaceae. *CtrMYB12a*/*b*/*c* belongs to subgroup 7 R2R3-MYB TFs and is related to Arabidopsis MYB12 which commonly regulate flavonol glycoside accumulation via the activation of *FLS* and shared flavonoid biosynthetic pathway genes (Stracke et al., 2007). In particular, *CtrMYB12a* shares differential expression patterns with *CtrFLS1/2* and *CtrCOMT1-4*. We predict that *CtrMYB12a* regulates flavonol glycoside accumulation by targeting *CtrFLS1/2* and *CtrCOMT1-4*. Conversely, *CtrMYB12c* does not share expression similarities with *CtrFLS1/2.* Instead, *CtrMYB12c* expression coincides with *CtrMYB2a*, suggesting that *CtrMYB12c* is more relevant to the regulation of anthocyanin than flavonol glycoside biosynthesis. In plants, the transcriptional activation strength of *FLS* are known to differ between orthologs of AtMYB12 (Mehrtens et al., 2005) but also associated paralogs of certain species (Shan et al., 2020). In *Freesia*, all four AtMYB12 orthologs FhMYBF1–4 positively regulated several shared flavonoid pathway genes and *FhFLS1* at different magnitudes when overexpressed, however FhMYBF4 lack the capacity to highly induce *FhFLS1* expression (Shan et al., 2020). Some studies have even shown that AtMYB12 orthologs potentially regulate the anthocyanin biosynthetic pathway genes (Czemmel et al., 2009; Zheng et al., 2019; Zhong et al., 2020).

CtrMYB32 and CtrMYB4a/b/c belong to subgroup 4 R2R3-MYB TFs which are active repressors that fine tune flavonoid levels by balancing the inductive effects of the MBW complex (Chen et al., 2019; LaFountain and Yuan, 2021). In particular, *CtrMYB32* and *CtrMYB4a* were consistently upregulated in *vmb* and *sflw* while the opposite was true for *CtrMYB4b* compared to *vyb* regardless of tissue type. Furthermore, *CtrMYB4a/b/c* showed significantly higher callus gene expression compared to the lamina in one or more stages post *vyb*. Since further anthocyanin pathway activation by the MBW complex does not benefit the establishment of color or contrast, in the callus, we predict that *CtrMYB4a/b/c* co-operatively repress the inductive effects of the MBW activity in *vmb* and *sflw* once the distinct black color has been established in *vyb.* Conversely, the weaker pathway repression in the labellum compared to the callus post *vyb* may signal the accumulation of anthocyanins.

### Implications and Future Directions

In the last decade, considerable progress has been made toward understanding the diversity and distribution (Peakall et al., 2010) and the regulation of the chiloglottones which underpin the sex pheromone mimicry of *Chiloglottis* orchids (Falara et al., 2013; Amarasinghe et al., 2015). However, the chemical and molecular basis of the floral pigmentation in *Chiloglottis*, and indeed of any Australian sexually deceptive orchids, has remained unknown. Here, we demonstrate that the black callus of *Chiloglottis trapeziformis* flowers is characterized by a high abundance of cyanidin-based anthocyanins and diverse flavonol glycoside co-pigments concentrated in a small, highly localized area. Conversely, a high abundance of flavonol glycoside co-pigments and low anthocyanin content is characteristic of the lamina. Differential expression of specific genes controlling the branchpoints into the respective anthocyanin (e.g., *DFR* and *LDOX*) and flavonol glycoside biosynthesis (e.g., *FLS*) and its subsequent modification toward methylated derivatives (e.g., *FMT*) is likely pivotal for generating the contrasting dark color of the callus against the green to reddish green lamina (Figure 3).

Beyond *Chiloglottis*, inconspicuous dull colored flowers with tissue-specific dark maroon to black pigmentation is common in sexually deceptive species. In Australia, several orchid genera have some sexually deceptive species which exhibit this tissue-specific contrast (e.g., within *Caladenia*, *Calochilus*), and in some genera, this is exclusively the case (e.g., *Drakaea*, *Cryptostylis*, *Paracaleana*, *Leporella*, *Caleana*) (Gaskett, 2011). Other examples of tissue-specific dark colors in sexually deceptive species outside Australia are the dark labella of many *Ophrys* species (Bradshaw et al., 2010) such as *Ophrys speculum* (Figure 1F), the maroon-black callus structure on the pink labellum of *Serapias lingua* (Vereecken et al., 2012; Pellegrino et al., 2017), the large elongated dark maroon labellum of Asian beetle-pollinated *Luisia teres* (Arakaki et al., 2016), the triangular-shaped dark maroon pigmentation on the labellum of bee-pollinated *Mormolyca ringens* (Singer et al., 2004), the central dark maroon labellum and column on an otherwise bright yellow corolla of *Telipogon peruvianus* (Martel et al., 2016, 2019), a combination of dark maroon-colored floral structures including antennae-like petals, anther, and labellum of south African beetle-pollinated *Disa forficaria* orchids (Cohen et al., 2021), and the raised black spots on the yellow to bright orange ray florets of some fly pollinated *Gorteria diffusa* (Ellis and Johnson, 2010) morphotypes (Figure 1G). In these cases, the dark pigmentation likely serves several key purposes important for pollination, including mimicking the color of female insects, providing strong chromatic and achromatic contrast, and aiding the exploitation of pollinator sensory biases.

All of these examples showcase a strong correlation between sexual deception and dark colored flowers across many different evolutionary lineages. As such, understanding the chemical and genetic mechanisms of how such dark coloration is attained is pivotal for understanding how sexual deception evolves. We predict that similar chemical constituents and/or spatiotemporal gene expression of underlying pathway genes (e.g., *DFR*, *LDOX*, and *FLS*) will be pivotal for dark, potentially contrasting tissue-specific pigmentation in other sexually deceptive species. Currently, *Ophrys* is the best-characterized genus to support this prediction. In some confirmed sexually deceptive *Ophrys* orchids such as *O. insectifera*, *O. sphegodes*, and *O. speculum* (Schiestl et al., 1999; Vignolini et al., 2012), cyanidin-based anthocyanins are the major floral anthocyanin pigment present in whole flowers or the labellum (Strack et al., 1989). Furthermore, the dark labellum color of *Ophrys speculum* (Figure 1F) was demonstrated to contain a blend of anthocyanin and quercetin flavonol glycoside co-pigments (Vignolini et al., 2012). Transcriptomic surveys of *O. exaltata*, *O. sphegodes*, *O. aymoninii*, and *O. garganica* (Sedeek et al., 2013; Piñeiro Fernández et al., 2019) reveal that anthocyanin and flavonol glycoside biosynthesis pathway genes are active in the dark labella of these species. In *Gorteria diffusa* that possess dark tissue-specific pigmentation flanked by brightly colored orange ray florets, it will be of interest to determine the types of floral pigments, cellular chemistry, and genetic mechanisms involved. In this latter case, the relevance of carotenoid (e.g., xanthophylls) or flavonoid (e.g., chalcones and aurones) pigment pathways are predicted to be the driver of the contrast.

Future studies will also be required to determine the key transcription factors involved and how they regulate the expression of key flavonoid biosynthetic, modification, and transport pathway enzymes in the flowers of *Chiloglottis* and other sexually deceptive plants. Members of the R2R3-MYB, bHLH, and WD-Repeat proteins, including those that we have briefly highlighted in this study, are prime candidates given that they are key determinants of anthocyanin pigmentation intensity and also patterning in many flowers (Davies et al., 2012; Wessinger and Rausher, 2012). However, MADS-box transcription factors should also be investigated as some are involved in establishing proper floral organ identity programs, photosynthesis, chlorophyll metabolism, and pigmentation in orchids (Pan et al., 2014; Hsu et al., 2021). The integration of various omics approaches (e.g., large-scale transcriptomic, metabolomic, ionomics) is also anticipated to provide a holistic view of how structural genes, regulatory factors, and cellular constituents (e.g., pigments, metal ions, pH) interact and mediate the evolutionary color transitions between sexually deceptive systems.

Our findings in *Chiloglottis trapeziformis* indicate that its floral color adaptations for sexual deception require complex tissue-specific coordinated regulation at multiple developmental stages, involving many genes and diverse pigment metabolic and cellular constituent regulating pathways. It is further of interest that the genus *Chiloglottis* belongs to a subtribe (Drakaeinae) in which all genera are exclusively sexually deceptive (with the exception of some rare self-pollinating cases) and all fall within a well-resolved clade on a long branch of the diverse predominantly Australasian tribe the Diurideae (Peakall et al., 2021). Thus, it is likely that the complex chemical and genetic basis of floral color mimicry may have been finely tuned by a long evolutionary process in these sexually deceptive orchids (Weston et al., 2014; Peakall et al., 2021). This is in contrast with some of the well-known flower color polymorphisms found within natural populations (Streisfeld and Rausher, 2011; Kellenberger et al., 2019), and even the flower color changes associated with key pollinator shifts that are often mediated by relatively simple genetic changes restricted to a few genes within the anthocyanin and flavonol glycoside pathways (Wessinger and Rausher, 2012). It will be of interest in future research to determine whether sexually deceptive lineages of more recent evolutionary origin than *Chiloglottis* (e.g., *Caladenia*, Caladeniinae; Diurideae) (Peakall et al., 2021) exhibit floral color adaptions based on simple genetic changes, as one might expect at the early stages in the evolution of sexual deception.

## Data Availability Statement

The datasets presented in this study can be found in online repositories. The names of the repository/repositories and accession number(s) can be found below: New sequence reads obtained for the sflw stage has been added to the existing BioProject accession PRJNA390683 and SRA study accession SRP1093281.

## Author Contributions

DW and JP performed the experiments. RP and DW secured the funding, designed the study, and coordinated the experiments and data analysis. DW wrote the article with assistance from RP and JP. All authors have read and approved the manuscript.

## Conflict of Interest

The authors declare that the research was conducted in the absence of any commercial or financial relationships that could be construed as a potential conflict of interest.

## Publisher’s Note

All claims expressed in this article are solely those of the authors and do not necessarily represent those of their affiliated organizations, or those of the publisher, the editors and the reviewers. Any product that may be evaluated in this article, or claim that may be made by its manufacturer, is not guaranteed or endorsed by the publisher.

## Footnotes

1
https://mona.fiehnlab.ucdavis.edu/

2
https://www.r-project.org/

3
https://transdecoder.github.io
