# Supplementary material for: Anthocyanin and Flavonol Glycoside Metabolic Pathways Underpin Floral Color Mimicry and Contrast in a Sexually Deceptive Orchid
Source: Front Plant Sci. 2022 Mar 23;13:860997. doi: 10.3389/fpls.2022.860997 (PMC8983864; doi:10.3389/fpls.2022.860997)
Supplement: Supplementary file 4 [file Data_Sheet_1.pdf]

Anthocyanin and flavonol glycoside metabolic pathways underpin floral colour mimicry and contrast in a sexually deceptive orchid

Darren C.J. Wong[1*], James Perkins[1], Rod Peakall[1]

[1]Ecology and Evolution, Research School of Biology, The Australian National University, Canberra 2600, Australia

*** Correspondence:**

Darren CJ Wong

darren.wong@anu.edu.au; wongdcj@gmail.com

+61 2 6125 9892

Keywords: ***Chiloglottis*****, anthocyanin, flavonol glycoside, flower, orchids, transcriptome, sexual deception (Min.5-Max. 8)**

SUPPLEMENTARY FIGURE

Figure S1. Developmental stage- and tissue-specific transcriptome analysis of *Chiloglottis trapeziformis* flowers. (A) Callus and labellum tissue transcriptome (6 samples, denoted with circles with a cross symbol) of mature sunflowers (*sflw*) obtained in this study were analysed alongside very young buds (*vyb*), very mature buds (*vmb*), and flower (*flw*) transcriptomes (31 samples) from earlier studies (Wong et al., 2017, 2018). Principal component analysis across 37 samples for the final set of 70,250 transcripts revealed a distinct separation of the tissues based on developmental stage (i.e. *vyb* vs *vmb*, *flw*, and *sflw* stages in PC1) followed by tissue type (i.e. separation of callus and labellum in PC2). (B) Differential expression analysis proceeded as follows: (i) four developmental stage comparisons between vmb (devC1/L1), sflw (devC2/L2) with vyb stage in the callus and labellum and (ii) three tissue comparisons between the callus versus labellum in respective vyb (ts1), vmb (ts2), and sflw (ts3) developmental stages. A summary of callus and labellum up- and down-regulated differentially expressed is indicated. (C) Summary of highly enriched (FDR < $1x10^{-5}$) Mapman BIN v4 functional categories describing generalized plant biological processes (BIN depth = 1) for upregulated (red) and downregulated (blue) genes of comparisons described in (B). Circle size and opacity represent the number of genes annotated in each enriched category and its associated enrichment score (represented as -$\log_{10}$FDR), respectively. Several highly enriched (FDR < 1 x 10-5) top-level BIN functional categories were identified in these comparisons (**Figure 1C** and **Supplementary Data 1**). For example, enrichment for carbohydrate metabolism (BIN3), cytoskeleton organisation (BIN20), and cell wall organisation (BIN21) were consistently observed in the downregulated genes while RNA processing (BIN16) and solute transport (BIN24) were in upregulated genes across the developmental stage comparisons regardless of tissue type (e.g. devC/L1 – 2). Conversely, tissue-

specific differences between callus and labellum tissues across four developmental stages (ts1 – 3) were commonly associated with an enrichment for lipid metabolism (BIN5) in upregulated genes while downregulated genes were enriched for photosynthesis (BIN1), phytohormone action (BIN11), and cell wall organisation (BIN21) pathways. Interestingly, closer inspection of lower-level BINs within the secondary metabolism.phenolics.flavonoid biosynthesis category (BIN9.2.2) revealed enriched terms (FDR < 1 x 10-2) related to chalcone metabolism (BIN9.2.2.1) were common in the upregulated genes of developmental stage and tissue comparisons while flavonol glycosides (BIN9.2.2.6) and aurones (BIN9.2.2.7) were common in downregulated genes. Conversely, flavanones (BIN9.2.2.2), dihydroflavonols (BIN9.2.2.4), flavonols (BIN9.2.2.5), and anthocyanidins (BIN9.2.2.9) were present in specific comparisons.

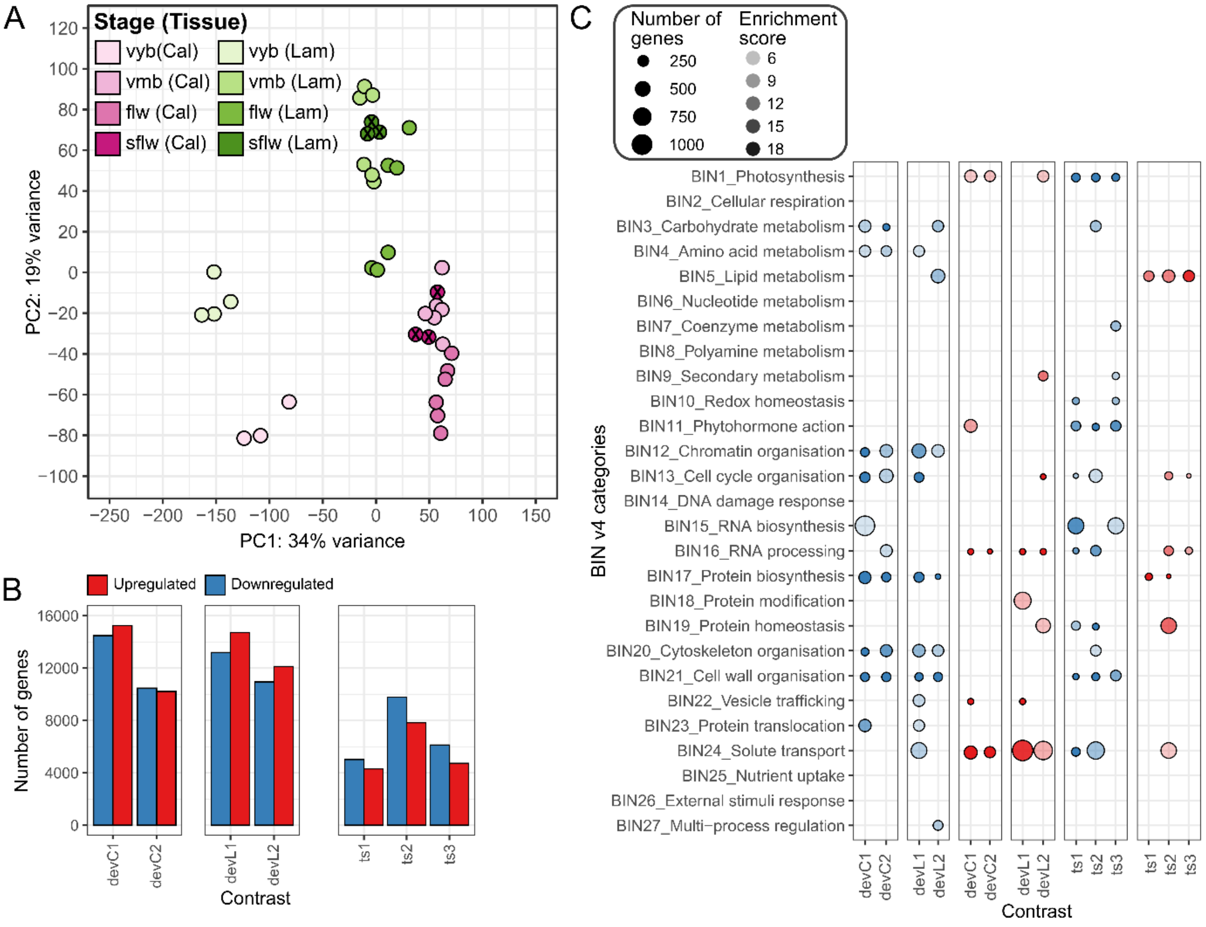

FIGURE S2. High-performance liquid chromatography- diode array detection-mass spectrometry of anthocyanins and flavonols in a representative extract of *Chiloglottis trapeziformis* very young bud (*vyb*) calli tissue. Absorbance spectra at 520nm and 365nm are depicted. Putative anthocyanins and flavonols are indicated. See **Table I** for additional details obtained from the ultra high-performance liquid chromatography-tandem mass spectrometry runs.

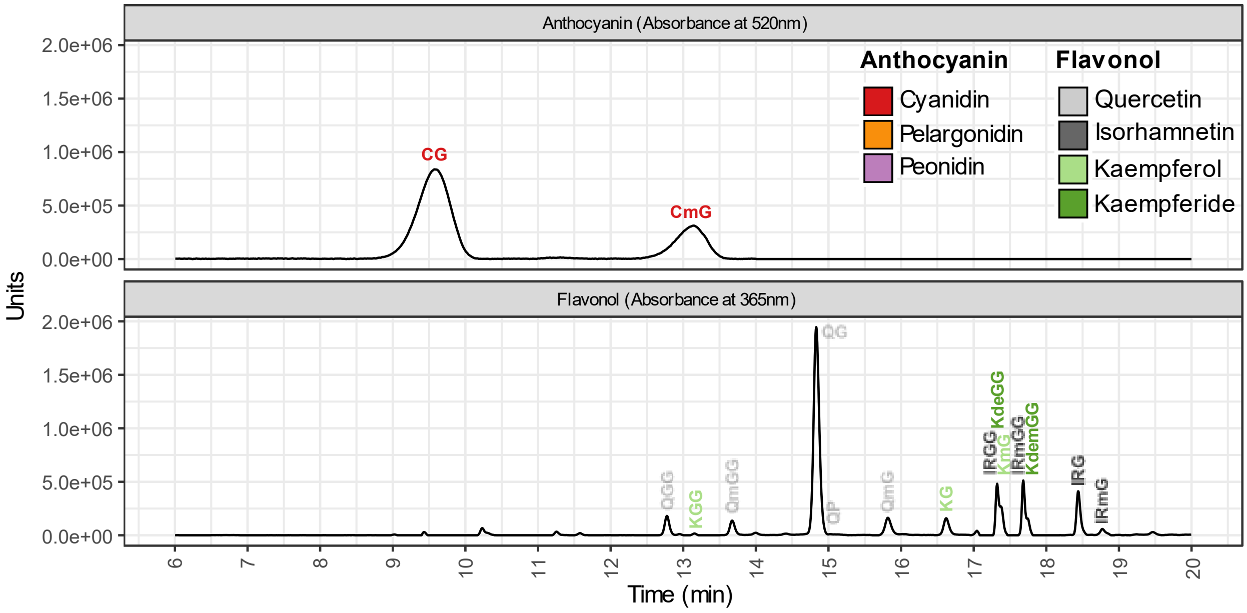

Figure S3. Hierarchical clustering of candidate anthocyanin and flavonol glucoside pathway-related gene expression in the calli and labellum lamina tissues of very young buds (*vyb*), very mature buds (*vmb*), and mature sunflowers (*sflw*). Associated clusters (labelled A, B, C, and D) of shared expression intensities (average $\log_2$ FPKM) are depicted to the left dendrogram of the clustered expression heatmap. Gene symbols and accompanying green, yellow, and purple square boxes indicate candidate flavonoid biosynthesis, modification, and transport genes prioritised in this study.

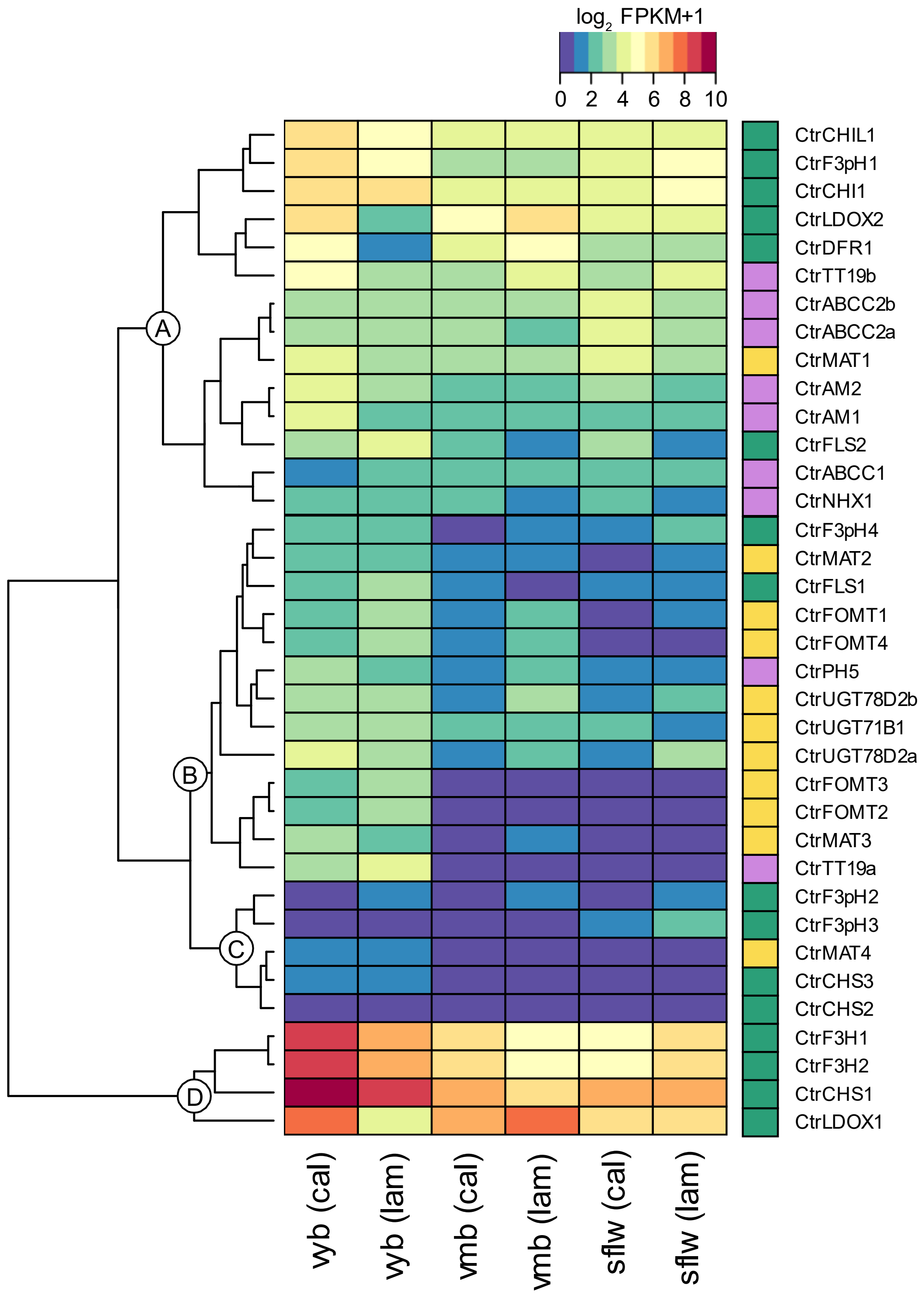

log2 FPKM+1
0
2
4
6
8
10
A
B
C
D
CtrCHIL1
CtrF3pH1
CtrCHI1
CtrLDOX2
CtrDFR1
CtrTT19b
CtrABCC2b
CtrABCC2a
CtrMAT1
CtrAM2
CtrAM1
CtrFLS2
CtrABCC1
CtrNHX1
CtrF3pH4
CtrMAT2
CtrFLS1
CtrFOMT1
CtrFOMT4
CtrPH5
CtrUGT78D2b
CtrUGT71B1
CtrUGT78D2a
CtrFOMT3
CtrFOMT2
CtrMAT3
CtrTT19a
CtrF3pH2
CtrF3pH3
CtrMAT4
CtrCHS3
CtrCHS2
CtrF3H1
CtrF3H2
CtrCHS1
CtrLDOX1
vyb (cal)
vyb (lam)
vmb (cal)
vmb (lam)
sflw (cal)
sflw (lam)

Figure S4. Hierarchical clustering of candidate anthocyanin and flavonol glucoside pathway-related differential expression patterns. Associated clusters (labelled 1, 2, 3, 4, and 5) of callus and labellum lamina differentially expressed (DE) pathway genes between *vyb* and *vmb* (devC1/L1), and *sflw* (devC2/L2) and callus vs labellum lamina tissue-specific DE genes between in *vyb* (ts1), *vmb* (ts2), and *sflw* (ts3) are depicted to the left dendrogram of the clustered $\log_2$ fold-change heatmap. Gene symbols and accompanying green, yellow, and purple square boxes indicate candidate flavonoid biosynthesis, modification, and transport genes prioritised in this study. Significant upregulation (FDR < 0.05, $\log_2FC > 0.5$) or downregulation (FDR < 0.05, $\log_2FC < -0.5$) in selected comparisons is indicated with an asterisk.

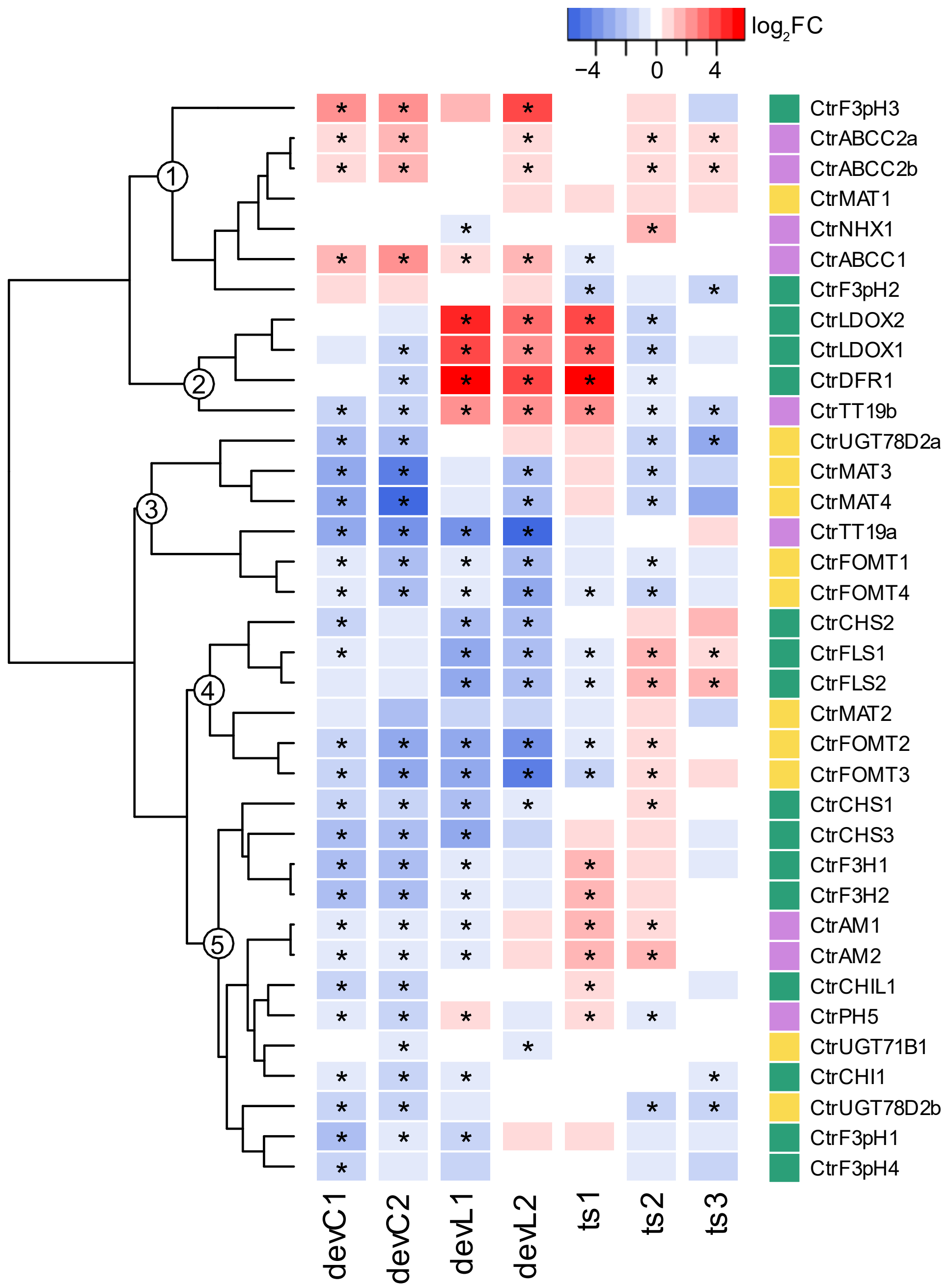
log2FC
−4
0
4
CtrF3pH3
CtrABCC2a
CtrABCC2b
CtrMAT1
CtrNHX1
CtrABCC1
CtrF3pH2
CtrLDOX2
CtrLDOX1
CtrDFR1
CtrTT19b
CtrUGT78D2a
CtrMAT3
CtrMAT4
CtrTT19a
CtrFOMT1
CtrFOMT4
CtrCHS2
CtrFLS1
CtrFLS2
CtrMAT2
CtrFOMT2
CtrFOMT3
CtrCHS1
CtrCHS3
CtrF3H1
CtrF3H2
CtrAM1
CtrAM2
CtrCHIL1
CtrPH5
CtrUGT71B1
CtrCHI1
CtrUGT78D2b
CtrF3pH1
CtrF3pH4
devC1
devC2
devL1
devL2
ts1
ts2
ts3
1
2
3
4
5

Figure S5. Developmental changes of anthocyanins and flavonol glycoside co-pigments in *Chiloglottis trapeziformis* flowers. Bar graph depict the individual (A) anthocyanin and (B) flavonol glycoside content (average ± s.e.) in the callus and labellum lamina of *Chiloglottis trapeziformis* flowers at different developmental stages (i.e. very young bud, *vyb*; young bud, *yb*; mature buds, *mb*; very mature bud, *vmb*; and naturally opened flowers in the field, *sflw*).

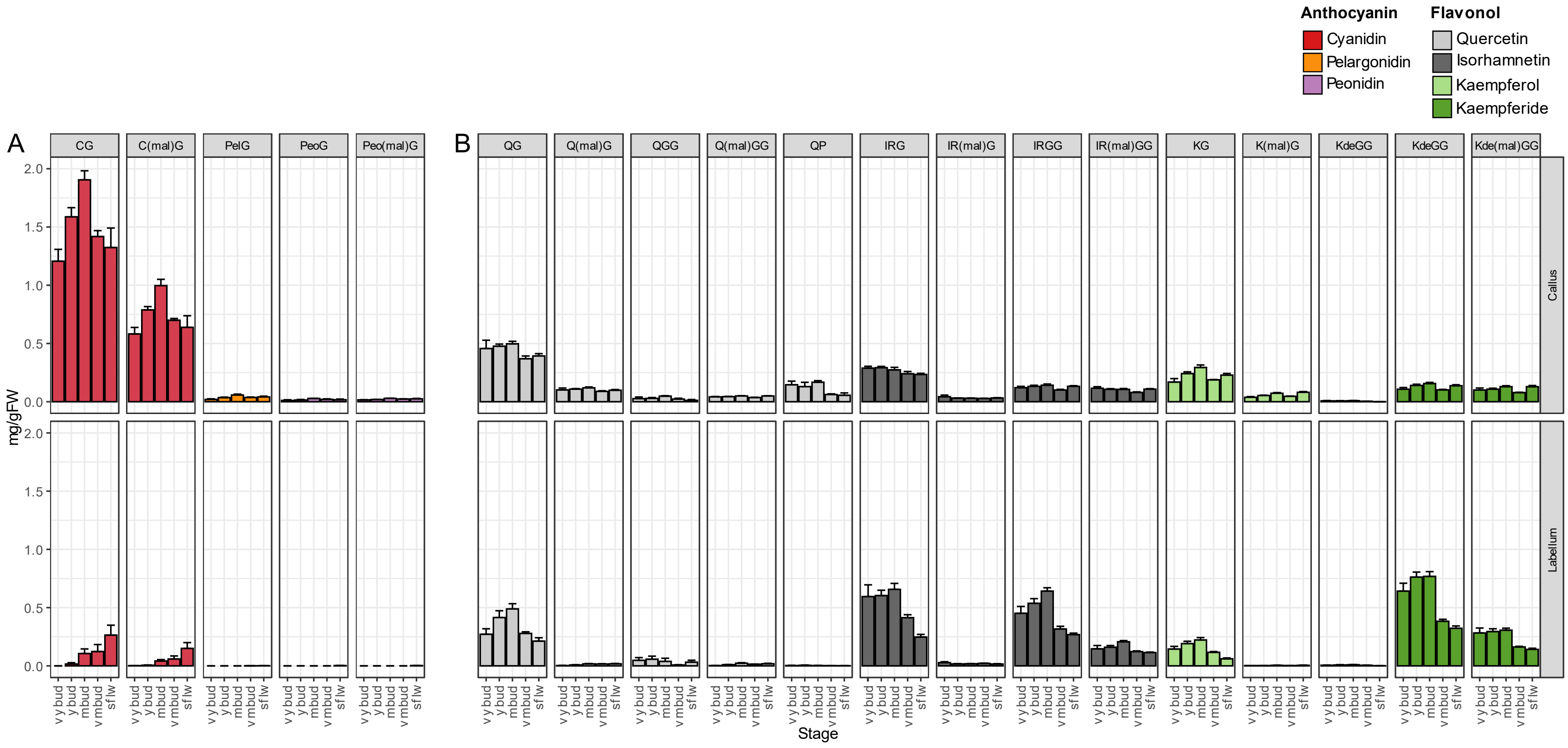

Figure S6. Developmental changes of flavonol glycoside co-pigments in *Chiloglottis trapeziformis* flowers. The bar graph depict the total flavonol glycoside content (average ± s.e.) according to their relevant aglycone (Q, quercetin; IR, isorhamnetin; K, kaempferol; Kde, kaempferide) in the callus and labellum lamina of *Chiloglottis trapeziformis* flowers at different developmental stages (i.e. very young bud, *vyb*; young bud, *yb*; mature buds, *mb*; very mature bud, *vmb*; and naturally opened flowers in the field, *sflw*).

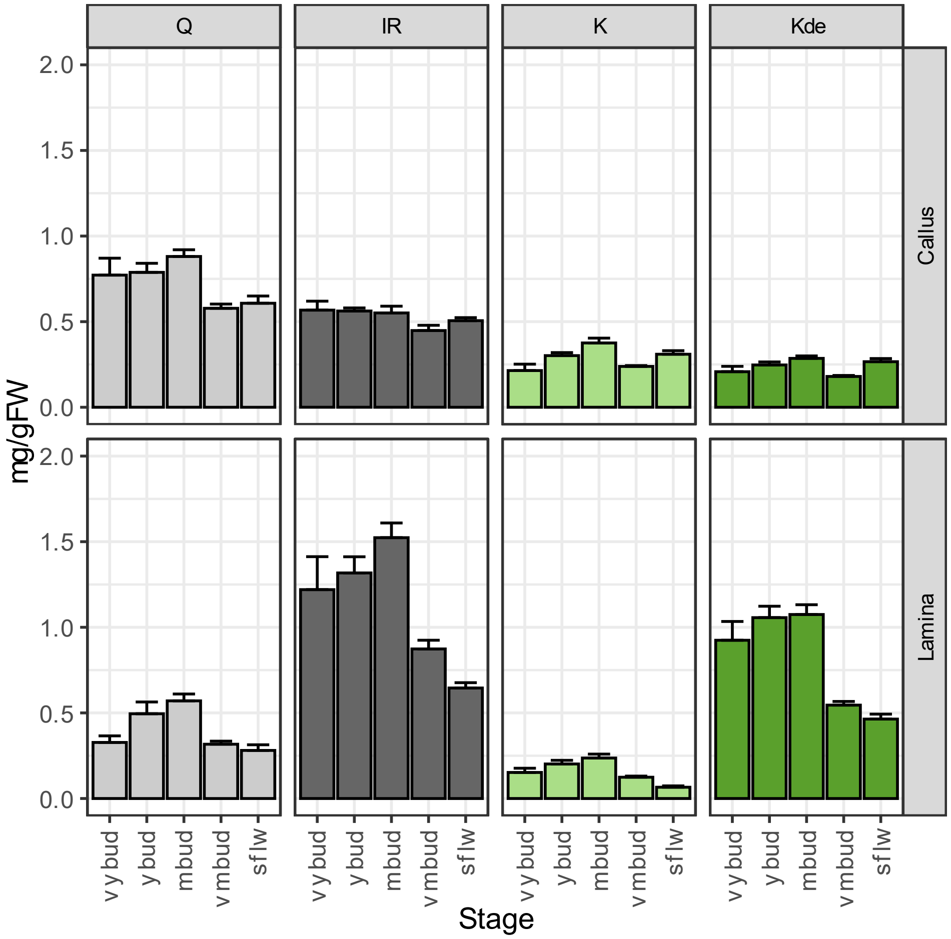

FIGURE S7. ANOVA and TukeyHSD test outcomes of total (**A** and **B**) anthocyanin and (**C** and **D**) flavonol glycoside content in the callus and labellum during flower development following Shapiro-Wilk test satisfying normality. Three samples of each tissue type at the *vyb* stage were analysed, and six for all other developmental stages were used, with the exception of the lamina at the *mb* stage for which only five samples were available.

A) Callus anthocyanin. ANOVA $F_{4,22}$ = 6.75, p < 0.001

```
> summary(ANOVA)
            Df Sum Sq Mean Sq F value  Pr(>F)
C_A$Stage    4  4.205  1.0512    6.75 0.00106 **
Residuals   22  3.426  0.1557
---
Signif. codes:  0 '***' 0.001 '**' 0.01 '*' 0.05 '.' 0.1 '

> TUKEY
  Tukey multiple comparisons of means
    95% family-wise confidence level

Fit: aov(formula = model)

$`C_A$Stage`
                      diff        lwr        upr     p adj
2_ybud-1_vybud   0.6097234 -0.2182174  1.4376643 0.2221110
3_mbud-1_vybud   1.1812772  0.3533364  2.0092181 0.0028401
4_vmbud-1_vybud  0.3633409 -0.4646000  1.1912817 0.6927388
5_vmbud-1_vybud  0.2103809 -0.6175599  1.0383218 0.9409550
3_mbud-2_ybud    0.5715538 -0.1044571  1.2475647 0.1249016
4_vmbud-2_ybud  -0.2463825 -0.9223934  0.4296284 0.8140368
5_vmbud-2_ybud  -0.3993425 -1.0753534  0.2766684 0.4244741
4_vmbud-3_mbud  -0.8179363 -1.4939472 -0.1419254 0.0127033
5_vmbud-3_mbud  -0.9708963 -1.6469072 -0.2948854 0.0026580
5_vmbud-4_vmbud -0.1529600 -0.8289709  0.5230509 0.9605555
```

B) Labellum anthocyanin. ANOVA $F_{4,21}$ = 3.71, p < 0.01

```
> summary(ANOVA)
            Df Sum Sq Mean Sq F value Pr(>F)
L_A$Stage    4 0.5952 0.14880   3.712 0.0195 *
Residuals   21 0.8419 0.04009
---
Signif. codes:  0 '***' 0.001 '**' 0.01 '*' 0.05 '.' 0.1 ' '
> TUKEY
  Tukey multiple comparisons of means
    95% family-wise confidence level

Fit: aov(formula = model)

$`L_A$Stage`
                      diff          lwr       upr     p adj
2_ybud-1_vybud  0.01973931 -0.402039324 0.4415180 0.9999073
3_mbud-1_vybud  0.14637411 -0.289236996 0.5819852 0.8519451
4_vmbud-1_vybud 0.18088407 -0.240894570 0.6026627 0.7071856
5_vmbud-1_vybud 0.41817871 -0.003599925 0.8399574 0.0527009
3_mbud-2_ybud   0.12663479 -0.234554852 0.4878244 0.8318875
4_vmbud-2_ybud  0.16114475 -0.183236061 0.5055256 0.6379781
5_vmbud-2_ybud  0.39843940  0.054058584 0.7428202 0.0183181
4_vmbud-3_mbud  0.03450996 -0.326679687 0.3956996 0.9984426
5_vmbud-3_mbud  0.27180460 -0.089385042 0.6329943 0.2034186
5_vmbud-4_vmbud 0.23729465 -0.107086171 0.5816755 0.2767192
```

C) Callus flavonol glycoside. ANOVA $F_{4,22}$ = 8.07 p < 0.0001

```
> summary(ANOVA)
            Df Sum Sq Mean Sq F value   Pr(>F)
C_F$Stage    4 1.3967  0.3492   8.066 0.000367 ***
Residuals   22 0.9523  0.0433
---
Signif. codes:  0 '***' 0.001 '**' 0.01 '*' 0.05 '.' 0.1 ' '
> TUKEY
  Tukey multiple comparisons of means
    95% family-wise confidence level

Fit: aov(formula = model)

$`C_F$Stage`
                      diff        lwr         upr     p adj
2_ybud-1_vybud   0.1373866 -0.2991073  0.57388058 0.8806261
3_mbud-1_vybud   0.3312840 -0.1052100  0.76777796 0.1983278
4_vmbud-1_vybud -0.3168239 -0.7533179  0.11967005 0.2340003
5_vmbud-1_vybud -0.0720613 -0.5085553  0.36443266 0.9875094
3_mbud-2_ybud    0.1938974 -0.1624985  0.55029320 0.5043645
4_vmbud-2_ybud  -0.4542105 -0.8106064 -0.09781471 0.0081833
5_vmbud-2_ybud  -0.2094479 -0.5658438  0.14694790 0.4295247
4_vmbud-3_mbud  -0.6481079 -1.0045037 -0.29171208 0.0001804
5_vmbud-3_mbud  -0.4033453 -0.7597411 -0.04694947 0.0214343
5_vmbud-4_vmbud  0.2447626 -0.1116332  0.60115843 0.2817848
```

D)Labellum flavonol glycoside. ANOVA $F_{4,21}$ = 21.28, p < 0.0001

```
> summary(ANOVA)
            Df Sum Sq Mean Sq F value   Pr(>F)
L_F$Stage    4 14.965   3.741   21.28 3.86e-07 ***
Residuals   21  3.693   0.176
---
Signif. codes:  0 '***' 0.001 '**' 0.01 '*' 0.05 '.' 0.1 '
> TUKEY
  Tukey multiple comparisons of means
    95% family-wise confidence level

Fit: aov(formula = model)

$`L_F$Stage`
                      diff        lwr        upr     p adj
2_ybud-1_vybud   0.4471620 -0.4361490  1.3304729 0.5687475
3_mbud-1_vybud   0.7813322 -0.1309474  1.6936118 0.1167939
4_vmbud-1_vybud -0.7631507 -1.6464617  0.1201602 0.1119663
5_vmbud-1_vybud -1.1674984 -2.0508093 -0.2841875 0.0060611
3_mbud-2_ybud    0.3341702 -0.4222521  1.0905925 0.6846059
4_vmbud-2_ybud  -1.2103127 -1.9315331 -0.4890923 0.0005197
5_vmbud-2_ybud  -1.6146604 -2.3358807 -0.8934400 0.0000122
4_vmbud-3_mbud  -1.5444829 -2.3009052 -0.7880606 0.0000441
5_vmbud-3_mbud  -1.9488306 -2.7052529 -1.1924083 0.0000015
5_vmbud-4_vmbud -0.4043477 -1.1255680  0.3168727 0.4722844
```

SUPPLEMENTARY TEXT

Candidate transcriptional activators and repressors of anthocyanin and flavonol glycoside pathway-related genes in *Chiloglottis trapeziformis*, in the context of relevant background literature

The developmental and tissue-specific transcriptional control of pigmentation and patterning is determined by the MBW protein complex. However, the precise component of this complex and how it regulates downstream genes that affect colouration varies between plants (Davies et al. 2012; Wessinger and Rausher 2012). Within the highly expanded R2R3-MYB TF family (Feller et al. 2011; Rodrigues et al. 2021), several subgroups are known to regulate various branches of the phenylpropanoid/flavonoid pathway among other functions. Notably, subgroup 5, 6, and 20 are often transcriptional activators that promote anthocyanin production while subgroup 7 promotes flavonol glycoside accumulation in various plants (Wong et al. 2016; Allan and Espley 2018; Rodrigues et al. 2021). Conversely, subgroup 4 members commonly act as active repressors of the anthocyanin and/or phenylpropanoid biosynthesis (LaFountain and Yuan 2021).

In this study of the contrasting labellum colours of the sexually deceptive orchid *Chiloglottis trapeziformis* , two subgroup 5, three subgroup 7, and four subgroup 4 R2R3-MYB TFs members were prioritised (**Figure 4, Supplementary Data S1 – 3**). CtrMYB2a/b belongs to subgroup 5 of R2R3-MYB TFs that contains several known orchid tissue-specific floral pigmentation regulators such as *Phalaenopsis* PeMYB2/11/12 (Hsu et al. 2015), *Rhyncholaeliocattleya* (*Rhyncholaelia* x *Cattleya*) Promoted Anthocyanin Pigmentation RcPAP1/2 (Li et al. 2020a), and *Oncidium* OgMYB1 (Chiou and Yeh 2008). Transient overexpression of PeMYB2 and RcPAP1/2 in white-flowered *P. aphrodite* ssp. *formosana* both induced the expression of endogenous flavonoid and anthocyanin biosynthesis genes (e.g. *F3H*, *F3'H*, *DFR*, or *ANS* genes) and the development of reddish-pink pigmentation (Hsu et al. 2015; Li et al. 2020a). In *Phalaenopsis*, *PeMYB2* is correlated with anthocyanin abundance in the petal/sepals of several cultivars and is highly expressed in full-red pigmented flowers. In *Rhyncholaeliocattleya* 'KOVA' flowers, *RcPAP1* is specifically expressed in the labellum of young flower buds while *RcPAP2* is expressed after flowering mainly in the sepal/petal.

Unlike subgroup 5 R2R3-MYBs of orchids, the role of other subgroups (e.g. 6, 7, 20, and 4) in the regulation of flavonoid metabolism and pigmentation remains poorly understood in the Orchidaceae. CtrMYB12a/b/c belongs to subgroup 7 of R2R3-MYB TFs (Mehrtens et al. 2005; Stracke et al. 2007; Sheehan et al. 2016; Yuan et al. 2016; Zheng et al. 2019; Shan et al. 2020) and is related to Arabidopsis MYB12 which commonly regulate flavonol glycoside accumulation via the activation of *FLS* and shared flavonoid biosynthetic pathway genes (Stracke et al. 2007). Interestingly, the transcriptional activation strength of *FLS* is known to differ between orthologs and/or paralogs of AtMYB12 in a wide range of species. For example, the *Zea mays* ortholog of AtMYB12, ZmP1 strongly activates the promoters of Arabidopsis *CHS* and *F3H* but showed poor induction for *FLS* (i.e. 18% of that observed for AtMYB12) (Mehrtens et al. 2005). In *Freesia*, all four AtMYB12 orthologs, FhMYBF1–4 positively regulated several shared flavonoid pathway genes and *FhFLS1* at different magnitudes when overexpressed, however, *FhMYBF4* lacked the capacity to highly induce *FhFLS1* expression (Shan et al. 2020). Some studies have

even shown that AtMYB12 orthologs potentially regulate dedicated anthocyanin biosynthetic pathway genes (Czemmel et al. 2009; Zheng et al. 2019; Zhong et al. 2020). For example, transient promoter assays revealed that the *LDOX* promoter is activated by VvMYBF1 in grapevine (Czemmel et al. 2009). In *Gerbera*, GhMYB1a failed to activate the *GhDFR* promoter, but significantly induced the expression of *GhDFR* and *GhANS* when overexpressed transiently in petals, indicating an indirect regulation of the anthocyanin biosynthetic pathway by GhMYB1a (Zhong et al. 2020).

CtrMYB32 and CtrMYB4a/b/c belongs to subgroup 4 R2R3-MYB TFs. It is well established that subgroup 4 R2R3-MYB TFs fine tune flavonoid levels in various plant tissues in response to developmental transitions or environmental cues by balancing the inductive effects of the MBW complex by repressing the promoter activities of the shared flavonoid and committed anthocyanin pathway genes (Chen et al. 2019; LaFountain and Yuan 2021). Interestingly, the expression of subgroup 4 R2R3-MYB TFs are often negatively correlated with anthocyanin accumulation, underlying biosynthetic genes, and positive pathway regulators functions. Consequently, they tend to to prevent ectopic accumulation of anthocyanins while those that are positively correlated, are often activated by the MBW complex, and provide feedback regulation. For example, *Freesia FhMYB27* efficiently repressed the inductive effects of the MBW components (FhPAP1 and FhTT8L) on the flavonoid and anthocyanin biosynthesis pathway genes and petal pigmentation when overexpressed in white *Freesia* flowers. Similarly, four peach flavonoid-related MYB repressors PpMYB17 – 20 abolished strong *DFR* promoter activation by the MBW components, PpMYB10.2 and PpbHLH3 (Zhou et al. 2016). Interestingly, *FhMYB27* is specifically expressed in the petal of red *Freesia* flowers and is strongly correlated with increasing anthocyanin content and expression of underlying pathway genes during the floral development (Li et al. 2020b). However, in pink peach flowers, both *PpMYB18/19* and *PpMYB17/20* showed distinct co-regulatory patterns with anthocyanin accumulation during flower development.

The bHLH TF family are also highly expanded in higher plants and classified into six major groups. One group (subgroup IIIf) is frequently implicated in the regulation of various branches of the phenylpropanoid/flavonoid pathway via protein-protein interactions with R2R3-MYB TFs (Feller et al. 2011; Davies et al. 2012). Unlike R2R3-MYB and bHLH TFs, WDR proteins are a rather small gene family with single genes often characterizing members of the anthocyanin-related TTG1/PAC1 clade in plants (Carey et al. 2004). Interestingly, genes encoding these positive pathway regulators are often constitutively expressed. This is often the case for homologs in some flowers. For example, petunia homologs of Arabidopsis GL3/EGL3 (PhJAF13) and TTG1 (*PhAN11*) often lack strong differential expression changes during flower development compared to key anthocyanin biosynthetic pathway genes (e.g. *PhDFR*) and R2R3-MYB activators and repressors (Albert et al. 2014). In *Clarkia gracilis* ssp. *sonomensis*, expression levels of *CgsbHLH2*, *CgsWDR1* and *CgsWDR2* homologs were similar in the distal/top region (pink) and the lower (white) cup region of the petal (Lin and Rausher 2021). In this study of *C. trapeziformis*, two putative subgroup IIIf bHLH TFs (CtrbHLH1) and one TTG1/PAC1 clade WDR protein (CtrTTG1) that are homologs of Arabidopsis GLABRA3 (GL3)/ ENHANCER OF GLABRA3 (EGL3) and TTG1, respectively (Zhang et al. 2003) were also identified.

Transcriptional regulation of pigmentation in the callus and lamina

In summary, homologs of twelve putative regulator genes (nine R2R3-MYB, two bHLH, and one WDR) and their gene expression patterns were highlighted in this study of *C. trapeziformis*. Perhaps most notable, among the patterns of gene expression was the finding that expression of *CtrMYB2a* and *CtrMYB12c* peaked in the callus of *vyb* and were 10 – 40-fold higher compared to the lamina. Conversely, *CtrMYB12a* was significantly upregulated (10-fold) in the lamina compared to callus of *vyb*. No other candidate genes showed tissue-specific differential expression in the *vyb*. Additionally, *CtrMYB12a* was upregulated in the callus from *vmb* onwards but remained consistent in the labellum compared to *vyb* while *CtrMYB2b* was upregulated post *vyb* regardless of tissue type. Compared to activator-type R2R3-MYBs, subgroup 4 R2R3-MYB repressors such as *CtrMYB32* and *CtrMYB4a* were consistently upregulated in *vmb* and *sflw* while *CtrMYB4b* and *CtrMYB4c* tended to decrease by the latter stages compared to *vyb* regardless of tissue type. Nonetheless, *CtrMYB4a*/*b*/*c* showed higher gene expression levels in the callus compared to the lamina in one or more stages post *vyb*. A lack of developmental stage and tissue-specific differential expression were generally observed for the two bHLH and one WDR candidate.

While beyond the scope of this present study, it will be of great interest in further research to more fully explore the patterns of gene expression in this candidate genes for transcriptional regulation, and to ultimately design transient expression studies to test further hypotheses on their role in the regulation of the pigmentation of flower colour in this and other sexually deceptive orchids.

REFERENCES

Albert NW, Davies KM, Lewis DH, Zhang H, Montefiori M, Brendolise C, Boase MR, Ngo H, Jameson PE, Schwinn KE (2014) A conserved network of transcriptional activators and repressors regulates anthocyanin pigmentation in Eudicots. Plant Cell 26:962–980 . https://doi.org/10.1105/tpc.113.122069

Allan AC, Espley R V. (2018) MYBs Drive Novel Consumer Traits in Fruits and Vegetables. Trends Plant Sci 23:693–705 . https://doi.org/10.1016/j.tplants.2018.06.001

Carey CC, Strahle JT, Selinger DA, Chandler VL (2004) Mutations in the pale aleurone color1 Regulatory Gene of the Zea mays Anthocyanin Pathway Have Distinct Phenotypes Relative to the Functionally Similar Transparent Testa Glabra1 Gene in Arabidopsis thaliana. Plant Cell 16:450–464 . https://doi.org/10.1105/tpc.018796

Chen L, Hu B, Qin Y, Hu G, Zhao J (2019) Advance of the negative regulation of anthocyanin biosynthesis by MYB transcription factors. Plant Physiol Biochem 136:178–187 . https://doi.org/10.1016/j.plaphy.2019.01.024

Chiou CY, Yeh KW (2008) Differential expression of MYB gene (OgMYB1) determines color patterning in floral tissue of Oncidium Gower Ramsey. Plant Mol Biol 66:379–388 . https://doi.org/10.1007/s11103-007-9275-3

Czemmel S, Stracke R, Weisshaar B, Cordon N, Harris NN, Walker AR, Robinson SP, Bogs J (2009) The grapevine R2R3-MYB transcription factor VvMYBF1 regulates flavonol synthesis in developing grape berries. Plant Physiol 151:1513–1530 . https://doi.org/10.1104/pp.109.142059

Davies KM, Albert NW, Schwinn KE (2012) From landing lights to mimicry: The molecular regulation of flower colouration and mechanisms for pigmentation patterning. Funct Plant Biol 39:619–638 . https://doi.org/10.1071/FP12195

Feller A, MacHemer K, Braun EL, Grotewold E (2011) Evolutionary and comparative analysis of MYB and bHLH plant transcription factors. Plant J 66:94–116 . https://doi.org/10.1111/j.1365-313X.2010.04459.x

Hsu C-C, Chen Y-Y, Tsai W-C, Chen W-H, Chen H-H (2015) Three R2R3-MYB transcription factors regulate distinct floral pigmentation patterning in *Phalaenopsis* spp. Plant Physiol 168:175–191 . https://doi.org/10.1104/pp.114.254599

LaFountain AM, Yuan YW (2021) Repressors of anthocyanin biosynthesis. New Phytol 231:933–949 . https://doi.org/10.1111/nph.17397

Li BJ, Zheng BQ, Wang JY, Tsai WC, Lu HC, Zou LH, Wan X, Zhang DY, Qiao HJ, Liu ZJ, Wang Y (2020a) New insight into the molecular mechanism of colour differentiation among floral segments in orchids. Commun Biol 3:1–13 . https://doi.org/10.1038/s42003-020-0821-8

Li Y, Shan X, Gao R, Han T, Zhang J, Wang Y, Kimani S, Wang L, Gao X (2020b) MYB repressors and MBW activation complex collaborate to fine-tune flower coloration in Freesia hybrida. Commun Biol 3:1–14 . https://doi.org/10.1038/s42003-020-01134-6

Lin RC, Rausher MD (2021) R2R3-MYB genes control petal pigmentation patterning in Clarkia gracilis ssp. sonomensis (Onagraceae). New Phytol 229:1147–1162 . https://doi.org/10.1111/nph.16908

Mehrtens F, Kranz H, Bednarek P, Weisshaar B (2005) The Arabidopsis transcription factor MYB12 is a flavonol-specific regulator of phenylpropanoid biosynthesis. Plant Physiol 138:1083–1096 . https://doi.org/10.1104/pp.104.058032

Rodrigues JA, Espley R V., Allan AC (2021) Genomic analysis uncovers functional variation in the C-terminus of anthocyanin-activating MYB transcription factors. Hortic Res 8:1–14 . https://doi.org/10.1038/s41438-021-00514-1

Shan X, Li Y, Yang S, Yang Z, Qiu M, Gao R, Han T, Meng X, Xu Z, Wang L, Gao X (2020) The spatio-temporal biosynthesis of floral flavonols is controlled by differential phylogenetic MYB regulators in Freesia hybrida. New Phytol 228:1864–1879 . https://doi.org/10.1111/nph.16818

Sheehan H, Moser M, Klahre U, Esfeld K, Dell'olivo A, Mandel T, Metzger S, Vandenbussche M, Freitas L, Kuhlemeier C (2016) MYB-FL controls gain and loss of floral UV

absorbance, a key trait affecting pollinator preference and reproductive isolation. Nat Genet 48:159–166 . https://doi.org/10.1038/ng.3462

Stracke R, Ishihara H, Huep G, Barsch A, Mehrtens F, Niehaus K, Weisshaar B (2007) Differential regulation of closely related R2R3-MYB transcription factors controls flavonol accumulation in different parts of the Arabidopsis thaliana seedling. Plant J 50:660–677 . https://doi.org/10.1111/j.1365-313X.2007.03078.x

Wessinger CA, Rausher MD (2012) Lessons from flower colour evolution on targets of selection. J Exp Bot 63:5741–9 . https://doi.org/10.1093/jxb/ers267

Wong DCJ, Schlechter R, Vannozzi A, H??ll J, Hmmam I, Bogs J, Tornielli GB, Castellarin SD, Matus JT (2016) A systems-oriented analysis of the grapevine R2R3-MYB transcription factor family uncovers new insights into the regulation of stilbene accumulation. DNA Res 23:451–466 . https://doi.org/10.1093/dnares/dsw028

Yuan YW, Rebocho AB, Sagawa JM, Stanley LE, Bradshaw HD (2016) Competition between anthocyanin and flavonol biosynthesis produces spatial pattern variation of floral pigments between Mimulus species. Proc Natl Acad Sci U S A 113:2448–2453 . https://doi.org/10.1073/pnas.1515294113

Zhang F, Gonzalez A, Zhao M, Payne CT, Lloyd A (2003) A network of redundant bHLH proteins functions in all TTG1-dependent pathways of Arabidopsis. Development 130:4859–4869 . https://doi.org/10.1242/dev.00681

Zheng J, Wu H, Zhu H, Huang C, Liu C, Chang Y, Kong Z, Zhou Z, Wang G, Lin Y, Chen H (2019) Determining factors, regulation system, and domestication of anthocyanin biosynthesis in rice leaves. New Phytol 223:705–721 . https://doi.org/10.1111/nph.15807

Zhong C, Tang Y, Pang B, Li X, Yang Y, Deng J, Feng C, Li L, Ren G, Wang Y, Peng J, Sun S, Liang S, Wang X (2020) The R2R3-MYB transcription factor GhMYB1a regulates flavonol and anthocyanin accumulation in Gerbera hybrida. Hortic Res 7: . https://doi.org/10.1038/s41438-020-0296-2

Zhou H, Peng Q, Zhao J, Owiti A, Ren F, Liao L, Wang L, Deng X, Jiang Q, Han Y (2016) Multiple R2R3-MYB transcription factors involved in the regulation of anthocyanin accumulation in peach flower. Front Plant Sci 7:1–11 . https://doi.org/10.3389/fpls.2016.01557
